# Solid Lipid Microparticles for Oral Delivery of Catalase: Focus on the Protein Structural Integrity and Gastric Protection

**DOI:** 10.1021/acs.molpharmaceut.0c00666

**Published:** 2020-07-30

**Authors:** Serena Bertoni, Daniele Tedesco, Manuela Bartolini, Cecilia Prata, Nadia Passerini, Beatrice Albertini

**Affiliations:** † PharmTech Lab, Department of Pharmacy and Biotechnology, University of Bologna, Via S. Donato 19/2, 40127 Bologna, Italy; ‡ Bio-Pharmaceutical Analysis Section (Bio-PhASe), Department of Pharmacy and Biotechnology, University of Bologna, Via Belmeloro 6, 40126 Bologna, Italy; § Biochemistry Lab, Department of Pharmacy and Biotechnology, University of Bologna, Via Irnerio 48, 40126 Bologna, Italy

**Keywords:** solid lipid microparticles, spray congealing, catalase, protein integrity, oral delivery, gastric inactivation

## Abstract

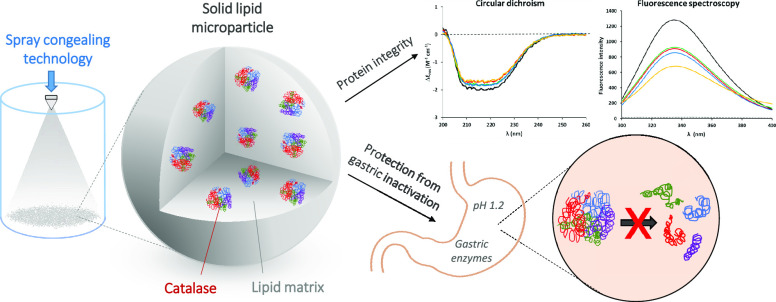

Protein inactivation either during the production process or along the gastrointestinal tract is the major problem associated with the development of oral delivery systems for biological drugs. This work presents an evaluation of the structural integrity and the biological activity of a model protein, catalase, after its encapsulation in glyceryl trimyristate-based solid lipid microparticles (SLMs) obtained by the spray congealing technology. Circular dichroism and fluorescence spectroscopies were used to assess the integrity of catalase released from SLMs. The results confirmed that no conformational change occurred during the production process and both the secondary and tertiary structures were retained. Catalase is highly sensitive to temperature and undergoes denaturation above 60 °C; nevertheless, spray congealing allowed the retention of most biological activity due to the loading of the drug at the solid state, markedly reducing the risk of denaturation. Catalase activity after exposure to simulated gastric conditions (considering both acidic pH and the presence of gastric digestive hydrolases) ranged from 35 to 95% depending on the carrier: increasing of both the fatty acid chain length and the degree of substitution of the glyceride enhanced residual enzyme activity. SLMs allowed the protein release in a simulated intestinal environment and were not cytotoxic against HT29 cells. In conclusion, the encapsulation of proteins into SLMs by spray congealing might be a promising strategy for the formulation of nontoxic and inexpensive oral biotherapeutic products.

## Introduction

The application of biological molecules (i.e., proteins, peptides, enzymes, nucleic acids, and hormones) as therapeutic agents has emerged over the past few decades as one of the most impactful areas of medicine.^1^ Biotherapeutics present substantial advantages for medical applications as a specific, selective, and efficient alternative to conventional drugs. Increasing efforts are thus dedicated to the development of biotherapeutics via an oral route, which is considered the most convenient way of drug administration. However, most therapeutic proteins have several stability issues. There are indeed a number of major hurdles that must be overcome to achieve a system able to successfully deliver an active biomolecule orally.^2^ The first challenge is represented by the formulation of the protein in a suitable delivery system, which should be nontoxic, biocompatible, and, more importantly, protein-friendly, i.e., it should not alter the protein function and structure. During the manufacturing of biopharmaceuticals, proteins are subjected to different forms of stress, such as agitation, temperature, light exposure, and oxidation,^3^ which can lead to protein denaturation, compromising the biological activity of the drug and the product quality. Secondly, the physiological conditions of the gastrointestinal tract (GIT), specifically the acidic pH and the digestive hydrolases of the gastric environment, can affect the protein structure, often leading to denaturation and activity loss. Consequently, bioactive proteins commonly need to be encapsulated to ensure protection during storage and after ingestion, as well as to allow the release at the appropriate site within the human body.^4^

Among the different methods of drug encapsulation, spray congealing (SC) has been attracting attention as it is a simple, low-cost, and solvent-free encapsulation technology.^5^ So far, few studies have investigated SC for the encapsulation of biological drugs. Maschke et al.^6^ demonstrated the feasibility of SC production of insulin-loaded microparticles, focusing on the influence of the process parameters (e.g., atomization pressure and spraying temperature) on the particle size and process yield, while Zaky et al.^7^ studied the distribution of fluorescently labeled bovine serum albumin (BSA) in the microsphere matrix and its impact on protein release. SC has been demonstrated to be an effective encapsulation technology even for high (10–20% w/w) protein loading, without affecting the protein structure.^8^

Furthermore, SC allows the production of microparticles using low-melting materials, such as long-chain solid lipids. These materials, specifically triglycerides, are receiving a great deal of attention due to their low cost, negligible toxicity, biodegradable properties, and versatility.^9^ The formulation of solid lipid microparticles (SLMs) for a successful oral delivery of biological drugs starts from the selection of proper lipid excipients with suitable hydrophobicity and tendency to undergo lipolysis. The first property is important to avoid the solubilization of SLMs in the first part of the GIT (oral cavity and stomach), allowing the retention of the encapsulated protein in the system to prevent its premature release and degradation. The second feature, however, is essential for the emulsification and digestion of the lipid matrix by bile salts and physiological lipases, allowing the release of the drug into the intestinal environment. Glyceryl trimyristate or trimyristin (Dynasan 114) has shown a good balance between those two properties and it is, therefore, suitable for the formulation of protein-loaded SLMs for oral administration.^10−13^ These studies confirmed that the intestinal release of the encapsulated proteins (β-galactosidase, lysozyme, and desmopressin) depends on a lipase-mediated degradation mechanism, which is related to the lipid composition and to the fed–fasted state conditions. However, there is little data about the ability of SLMs to retain the incorporated protein after exposure to gastric media. The protection of proteins from gastric degradation by multiparticulate formulations has been mostly studied for polymeric systems based on natural^14^ or synthetic pH-responsive^15,16^ polymers. Few studies have focused on systems based on lipid excipients, despite their multiple advantages as carriers, such as biodegradability and the absence of toxicity. Specifically, the research in the field has been limited to lipid-based nanosystems: solid lipid nanoparticles, nanoemulsions, liposomes, and nanocapsules.^17^ These studies highlighted the role of particle composition on the protein protection from degradation.^18^ One of our recent works^10^ showed that SLMs based on a fatty acid (myristic acid) were unable to retain the integrity of lactase, compared to those prepared with the corresponding triglyceride (glyceryl trimyristate). However, no study has explored the protection ability of SLMs containing other glycerides, despite their promising properties. Moreover, the ability of Dynasan 114-based SLMs to prevent gastric inactivation of the encapsulated protein did not exceed 70%.^10^ Therefore, other glyceride-based formulations need to be explored as potential carriers for the oral delivery of proteins.

This study aims to encapsulate a model protein, catalase (CAT), in spray congealed glyceride-based SLMs to evaluate the potential of these delivery systems for the oral administration of protein drugs. Specifically, the protein activity before and after encapsulation was assayed to assess the feasibility of the SC process in the production of different SLMs. Changes in the protein integrity after encapsulation and possible interactions with the carrier were studied by means of various techniques. Specifically, differential scanning calorimetry (DSC), Fourier-transform infrared (FT-IR) spectroscopy, and Raman spectroscopy were used for the study of CAT-loaded SLMs at the solid state, whereas circular dichroism (CD) and fluorescence spectroscopies were used for the analysis of CAT solutions after release from particles. Moreover, the ability to protect the biological compound from gastric inactivation was investigated, with a focus on the retention of catalytic activity after gastric transit.

## Materials and Methods

### Materials

Catalase from bovine liver (activity: 2000–5000 units/mg solid; 527 residues, molecular weight (MW): 59.92 kDa; used as received), ammonium molybdate (AM), and hydrogen peroxide solution were purchased from Sigma-Aldrich (Steinheim, Germany). Pepsin from porcine gastric mucosa (tested according to European Pharmacopoeia (Ph. Eur.)) and lipase from *Rhizophus niveus* (Lipase RN, ≥1.5 U/mg) were purchased from Sigma-Aldrich (Steinheim, Germany). Lipase RN can be used for in vitro lipid digestion as, like human gastric lipase, it is active on triglycerides with an optimum pH range of 5–7.^19^ Glyceryl monostearate was supplied by Prabo srl (Cremona, Italy). Precirol ATO 5 (glyceryl distearate) was kindly donated by Gattefossè (Milan, Italy). Dynasan 114 (trimyristin), Dynasan 116 (tripalmitin), and Dynasan 118 (tristearin) were obtained from Sasol (Witten, Germany). All other chemicals were of analytical grade. The colon cancer cell line HT29 was purchased from the American Type Culture Collection (Manassas, VA). For cell culture, Roswell Park Memorial Institute (RPMI) 1640 medium was obtained from Labtek Eurobio (Milan, Italy), fetal calf serum from Euroclone (Milan, Italy), RPMI 1640 medium, l-glutamine, and methylthiazolyldiphenyl-tetrazolium bromide (MTT) were purchased from Sigma-Aldrich (St. Louis, MO).

### CAT Activity Assay

The activity of the free enzyme was determined by a spectrophotometric assay based on the reaction of undecomposed hydrogen peroxide with AM to produce a yellow complex, characterized by an absorption maximum at 410 nm.^20,21^ First, 50 μg/mL enzyme solution in phosphate buffer (50 mM, pH 7.0) and substrate solution (H_2_O_2_, 125 mM in phosphate buffer 50 mM, pH 7.0) were prepared and stored at 4 °C before use. The reaction was started by adding the enzyme solution (0.1 mL) to 0.4 mL of substrate solution. After 0.5 min at 37 °C, the reaction was stopped by the addition of 2 mL of AM solution (32.4 mM in water). The yellow complex formed by the reaction of AM with the unreacted H_2_O_2_ was left to develop for 5 min before measuring the absorbance at 410 nm. A picture of the yellow complex formed and the calibration curve of the complex (*R*^2^ = 0.9993) are shown in Figure S1, Supporting Information (SI). Based on the calibration curve of the complex, the molar amount of decomposed H_2_O_2_ in the reaction mixture was determined and the activity of CAT (*U*, expressed in μmol/min) was calculated using eq 1, adapted from He et al.:^22^
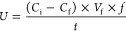
1where *C*_i_ (μM) is the initial concentration of the substrate, *C*_f_ (μM) is the concentration of the substrate after stopping the reaction, *V*_f_ (L) is the volume of the incubation mixture after termination, *f* is the total dilution factor of the sample, and *t* (min) is the incubation time. CAT activity was defined as the amount of enzyme that decomposes 1 μmol of H_2_O_2_/min under standard assay conditions (100 mM H_2_O_2_, pH 7, and 37 °C).

### Preparation of Solid Lipid Microparticles (SLMs)

Five formulations of SLMs were produced by spray congealing using a wide pneumatic nozzle (WPN) atomizer (Table 1). The excipient was heated up to a temperature 5 °C above its melting point, then raw CAT was added as powder (5 or 20% w/w) into the melted carrier, and magnetically stirred to obtain a stable suspension, which was loaded into the feeding tank. The temperature of the nozzle was set to 5 °C above the melting point of the carrier, whereas the inlet air pressure of the spray was set at 1.5 bar for all the formulations. The atomized molten droplets hardened during the fall into a cylindrical cooling chamber, which was held at room temperature (25 °C). Finally, SLMs were collected from the bottom of the cooling chamber and stored in polyethylene closed bottles at 25 °C. The product yield (% w/w) was determined by dividing the quantity of SLMs recovered by the amount of CAT and lipid loaded into the spray nozzle (total batch size).^8^

**Table 1:** Composition of CAT-Loaded SLMs

	constituents (%, w/w)				
SLMs samples	glyceryl trimyristate (Dynasan 114)	glyceryl tristearate (Dynasan 118)	glyceryl distearate (Precirol ATO 5)	glyceryl monostearate	API^a^
F1	95				5
F2	47.5	47.5			5
F3	47.5		47.5		5
F4	47.5			47.5	5
F5	80				20

a The theoretical amount of protein loaded corresponded to 50 and 200 μg/mg SLMs for 5 and 20% w/w active pharmaceutical ingredient (API)-loaded SLMs, respectively.

### SLMs Characterization

#### Size and Morphology

The size distribution of the SLMs was evaluated by sieve analysis, using a vibrating shaker (Octagon Digital, Endecotts, London, U.K.) and seven standard sieves (Scientific Instruments, Milan, Italy) of 50, 100, 150, 200, 250, 355, and 500 μm. The shape and surface morphology of SLMs were assessed by means of scanning electron microscopy (SEM). Samples were fixed on the sample holder with double-sided adhesive tape and examined by means of a scanning electron microscope (ESEM Quanta 200, FEI Company, Oxford Instruments) operating at 10.0 kV accelerating voltage.

#### Hot Stage Microscopy (HSM) Analysis

Physical changes in the samples during heating were monitored by HSM using a hot stage apparatus (Mettler-Toledo S.p.A., Novate Milanese, Italy) mounted on a Nikon Eclipse E400 optical microscope connected to a Nikon digital net camera DN100 for image acquisition. The magnification was set at 10×. The samples were equilibrated at 25 °C for 1 min and heated at a scanning rate of 10 °C/min in the desired ranges of temperature.

#### Differential Scanning Calorimetry (DSC)

DSC measurements were performed using a PerkinElmer DSC 6 (PerkinElmer, Beaconsfield, U.K.). The calibration of the instrument was performed with indium and lead for the temperature, and with indium for the measurement of the enthalpy. The samples, weighing 6–10 mg, were placed into the DSC under a nitrogen flux (20 mL/min) and heated from 25 to 150 °C at a scanning rate of 10 °C/min.

### Structural Integrity of CAT after Encapsulation

#### Raman Spectroscopy

Raman spectra were acquired on a micro-Raman Renishaw InVia spectrometer equipped with a Leica DMLM microscope. The excitation source was a diode laser with a wavelength of 780 nm adjusted to a power of 15 mW. Raman spectra were acquired from 100 to 3600 cm^–1^ using an objective with 50× magnification. The spectra were collected with an exposure of 10 s and four accumulations. To improve readability, the baseline was subtracted using Renishaw WiRE 2.0 software (cubic spline interpolation).

#### FT-IR Spectroscopy

Infrared spectra were recorded by a Jasco FT/IR-4200 IR spectrometer (Milan, Italy) using the KBr disc method. The samples were mixed with KBr and compressed into tablets (10 mm in diameter and 1 mm in thickness) using a manual hydraulic tablet presser (PerkinElmer, Norwalk). The scanning range was set to 650–4000 cm^–1^ and the resolution was set to 1 cm^–1^.

#### Circular Dichroism (CD) Spectroscopy

The structural integrity of CAT released from the SLMs in a simulated intestinal medium was also studied. Two hundred milligrams of SLMs was added to 10 mL of phosphate buffer, pH 6.8 and kept under agitation (250 rpm) at 37 °C for 2 h. The solution was then centrifuged (5585*g*, 10 min) and the supernatant was filtered (0.22 μm nylon filters) to eliminate the residual lipid carrier. The solutions of CAT obtained were stored in a refrigerator and analyzed within the same day. Then, the secondary structure of CAT was evaluated by CD analysis in the far-UV (260–200 nm) spectral range. CAT samples, as extracted from SLMs (F1–F4) in phosphate buffer pH 6.8, were preliminarily quantified based on the absorbance at 280 nm (ε_280_ = 64 290 M^–1^ cm^–1^) and eventually diluted to 10 μM before being submitted to far-UV CD analysis. CD measurements were carried out on a Jasco J-810 spectropolarimeter (Tokyo, Japan) using a 0.5 mm QS quartz cell (Hellma Analytics, Milan, Italy), a 2 nm spectral bandwidth, a 20 nm/min scanning speed, a 2 s data integration time, a 0.2 nm data interval, and an accumulation cycle of three runs per spectrum. The far-UV CD spectra of CAT samples were blank-corrected, converted to molar units per residue (Δε_res_, in M^–1^ cm^–1^), and compared with the CD spectrum of standard CAT (10.5 μM in phosphate buffer pH 6.8).

#### Fluorescence Spectroscopy

To confirm the stability of the tertiary structure, samples of CAT released from SLMs were analyzed by fluorescence spectroscopy. Samples of CAT released from SLMs were prepared following the same procedure used for CD analysis and analyzed by fluorescence spectroscopy. Fluorescence emission spectra were recorded by a Jasco FP-750 spectrofluorometer (Tokyo, Japan) between 300 and 400 nm with an excitation wavelength of 280 nm.

### Evaluation of CAT Activity after Encapsulation

To measure the activity of SLM-loaded CAT, the lipid matrix had to be dissolved and the enzyme released. Preliminary experiments were performed to find the suitable solvent to dissolve the SLMs without influencing CAT activity. An accurately weighed amount of SLMs (around 15 mg) was dissolved in dichloromethane (DCM, 0.4 mL), which was able to dissolve the lipid carrier but not the enzyme powder. After complete solubilization of the SLMs, 10 mL of phosphate buffer (50 mM, pH 7) was added, and the two immiscible phases were shaken to allow the solubilization and diffusion of the highly water-soluble CAT into the aqueous phase. Then, a 0.1 mL aliquot of this solution was used for the activity assay. Tests were performed on free CAT and on physical mixtures (free CAT mixed with unloaded SLMs) to verify the efficiency of the extraction process. Recovery values of 99.85 ± 1.61 and 95.33 ± 2.80% for free CAT and on the physical mixture were achieved, respectively. The activity of encapsulated CAT is given as U/mg SLMs.

### Determination of Protein Content

To measure the total protein content, the optimized procedure for CAT released from SLMs described in the section “Evaluation of CAT Activity after Encapsulation” was employed. After complete solubilization of the SLMs and addition of the 10 mL of phosphate buffer (50 mM, pH 7), the aqueous phase was assayed spectrophotometrically (Cary 60 UV–vis spectrometer, Agilent Technologies GmbH, Waldbronn, Germany) at 280 nm.^23^ Each formulation was analyzed in triplicate and the results were expressed as mean ± standard deviation (S.D.).

### Stability of CAT after Exposure to Simulated Gastric Conditions

To mimic the transit of SLMs through the stomach, the particles were incubated in simulated gastric conditions and the CAT activity was determined afterward. Specifically, two factors were evaluated: the extremely acidic pH of the stomach and the digestive hydrolases. For the former, a HCl solution (pH 1.2) was used. For the latter, the two main gastric digestive enzymes (pepsin and gastric lipase) were added to a buffer^24^ (68 mM NaCl, 2 mM Tris, 2 mM maleic acid) corrected to pH 4.5 with HCl. A pH of 4.5, an intermediate between the optimum pHs of pepsin (2–4) and lipase RN (5–7) and consistent with the pH of the fed stomach during digestion, was selected.^19,25^ Concentrations of enzymes were chosen to maintain activity levels similar to those observed in the fed human stomach, i.e., 30 U/mL pepsin^26^ and 40 U/mL gastric lipase.^19^ SLMs were incubated for 1 h in 20 mL of the medium under moderate agitation (250 rpm) at 37 °C. After incubation, SLMs were filtered, washed with water to eliminate the gastric media, and dried overnight. Then, the activity of the loaded CAT was measured.

### In Vitro Release Studies

In vitro release studies were carried out simulating the transit through the GIT. Samples of 65–70 mg of SLMs were dispersed in 40 mL of simulated gastric fluid (HCl solution, pH = 1.2) and incubated at 37 °C under magnetic stirring for 1 h. Then, the pH was adjusted to 6.8 using a 0.5 M Na_2_HPO_4_ solution to simulate the passage of the SLMs to the intestine and the test was continued for 4 h. Aliquots (1.0 mL) were withdrawn from the dissolution medium at predetermined time intervals (30, 60, 70, 90, 120, 150, 180, 240, 300 min) and replaced with fresh medium; 0.1 mL was used for the activity assay.

### MTT Assay on HT29 Cell Culture

MTT viability assay was performed to assess the biocompatibility of SLMs with intestinal cells. The human colon adenocarcinoma intestinal cell line (HT29), kindly provided by Prof. Natalia Calonghi (University of Bologna), was grown in RPMI 1640 medium supplemented with 10% fetal calf serum and 2 mM glutamine at 37 °C and 5% CO_2_. HT29 cells were seeded at 2 × 10^4^ cells/cm^2^ in a plastic well (60 cm^2^) and exposed to treatments after 1 day from the seeding. Cells (2 × 10^4^/cm^2^) were incubated with different concentrations (50–2000 μg/mL) of unloaded SLMs and CAT-loaded SLMs (diameters between 100 and 200 μm) for 24 h at 37 °C. The cells were then incubated with 5 mg/mL MTT for 4 h at 37 °C. Purple formazan salt crystals, formed during cell incubation, were dissolved by adding the solubilization solution (10% SDS, 0.01 M HCl). Plates were incubated overnight in a humidified atmosphere (37 °C, 5% CO_2_) and the absorbance was measured in a multiwell plate reader (Wallac Victor2, PerkinElmer) at 570 nm.

### Statistical Analysis

All results were expressed as mean ± standard deviation (S.D.). One-way analysis of variance (ANOVA) followed by the Bonferroni post hoc test (GraphPad Prism, GraphPad Software Inc., CA) was used to analyze the data and the level of significance was set at the probabilities of *p < 0.05, **p < 0.01, and ***p < 0.001.

## Results and Discussion

The development of active oral biotherapeutics is not simple and requires a careful consideration of the physicochemical properties of the encapsulated protein (e.g., molecular weight, hydrophobicity, stability to different pHs, temperature, and solvents).^27^ CAT is a tetrameric enzyme consisting of four identical subunits of 500 amino acids (ca. 60 kDa) each, plus four groups of porphyrin heme (iron) to enable its catalytic activity.^28^ Due to the ability to convert hydrogen peroxide (H_2_O_2_) to water and molecular oxygen, CAT has a key role in the protection from oxidative stress.^29^ CAT has been explored as a therapeutic agent for its antioxidant activity.^28,30,31^ Several factors, related to the preparation process as well as to the physiological environment once administered, can affect the protein structure and function. Free CAT characterization data (kinetic analysis, influence of pH and temperature on CAT activity) are reported in the Supporting Information. Since CAT activity was significantly reduced at high temperatures and low pH values (Figure S2, SI), it is of fundamental importance, in view of an oral administration, to develop a system able to protect the enzyme from the gastric environment, where the pH is extremely low. Moreover, it is important to avoid low pH values in the solubilization process and/or heating of CAT solutions above room temperature.

Considering these limitations, SC can be evaluated as a suitable technology for CAT encapsulation because it allows the encapsulation of the active ingredient at the solid state without the use of aqueous or organic solvents, which are usually required when a double emulsion solvent evaporation method is used. In SC, the active ingredient can be employed directly as a lyophilized powder, if available in this form, with important advantages in terms of stability during particle production and storage. On the other hand, SC requires the heating of the carrier to its melting temperature, although the exposure time to high temperatures of the active ingredient during the SC process is generally short as, after addition of the API to the melted carrier, the mixture solidifies immediately upon atomization. Specifically, on comparing with other heating-based methods used for the preparation of lipid microparticles (e.g., melt emulsification method), where a hot mixture is vigorously homogenized and then cooled to room temperature,^32^ SC allows more gentle mixing and shorter exposure times to high temperatures.

### SLMs Characterization

Five different SLMs formulations were produced (Table 1). CAT was first encapsulated at 5% w/w and Dynasan 114 was used as the excipient (F1). Then, mono-, di- and triglycerides with longer hydrophobic chains (C18), which are less subject to lipolysis than glyceryl trimyristate (C14),^10^ were added to the SLM composition at a 1:1 weight ratio (F2–F4). Additionally, a formulation with a higher CAT loading (20% w/w) (F5) was evaluated.

**Figure 1:**
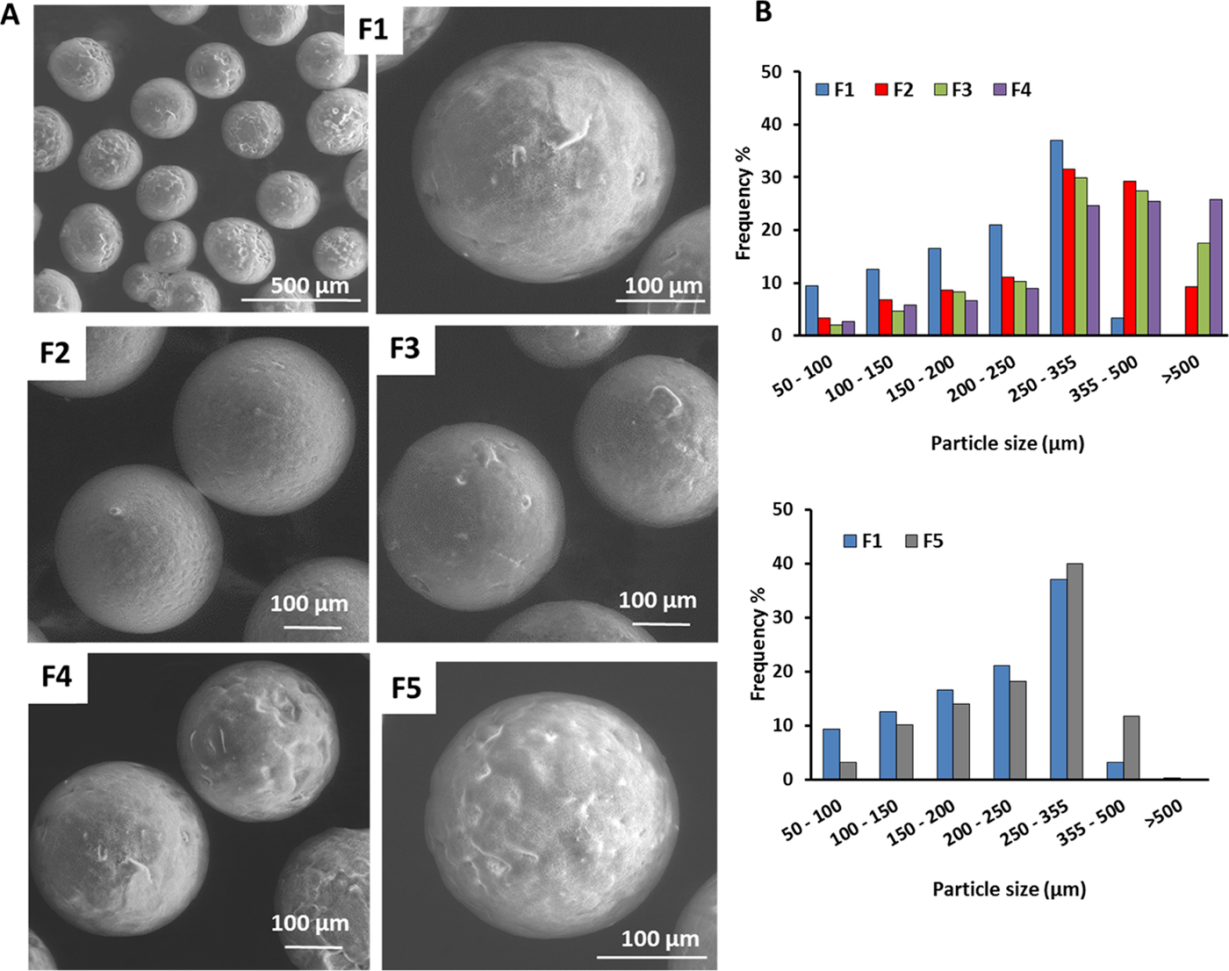
SEM images of F1 (at two different magnifications), F2, F3, F4, and F5 SLMs (A) and particle size distribution of SLMs (B).

The SEM images of the SLMs, reported in Figure 1A, showed nonaggregated particles with a spherical shape. Imperfections on the particle surface were probably due to the carrier morphology after the SC process, as is more evident in F1, F4, and F5 SLMs compared to F2 and F3 SLMs. CAT solid particles were not observed on the surface of SLMs, neither with 5% (F1–F4) nor with 20% (F5) of CAT. Particle size analysis (Figure 1B) revealed that SLMs had Gaussian distributions and dimensions in accordance with those observed by SEM. F1 SLMs had diameters ranging between 50 and 500 μm, with the main particle size fraction within 250 and 355 μm. Changes in the composition influenced the particle size and, in particular, the diameter of SLMs tended to increase in the formulations F2, F3, and F4, where the fraction of particles >500 μm was consistent. Thus, the addition of stearate glycerides led to the production of bigger particles, probably due to a higher viscosity of the molten excipient in the case of a binary mixture with myristate and stearate glycerides. Differently, changes in drug loading did not have a marked influence on the particle size, as F1 and F5, prepared exclusively with Dynasan 114, showed similar size distributions (Figure 1B).

Particle size is an important parameter in the development of an oral drug delivery system. For example, the release properties of SLMs are deeply influenced by their particle size. Generally, drug release is faster from smaller particles compared to bigger ones, a behavior observed both for polymeric^33,34^ and lipid^35^ microparticles. A recent study has evidenced that the release behavior of large SLMs (>250 μm) is less affected by the properties of the dissolution media (such as viscosity and the presence of surfactants as bile salts and lecithin) and more dependent on the lipid excipient compared to that of small SLMs.^35^ In addition, the particle size can impact on the ability to protect the encapsulated drug from the external environment. In this study, SLMs with diameters between 250 and 355 μm, i.e., the prevalent size fraction, were selected for all of the further experiments.

**Figure 2:**
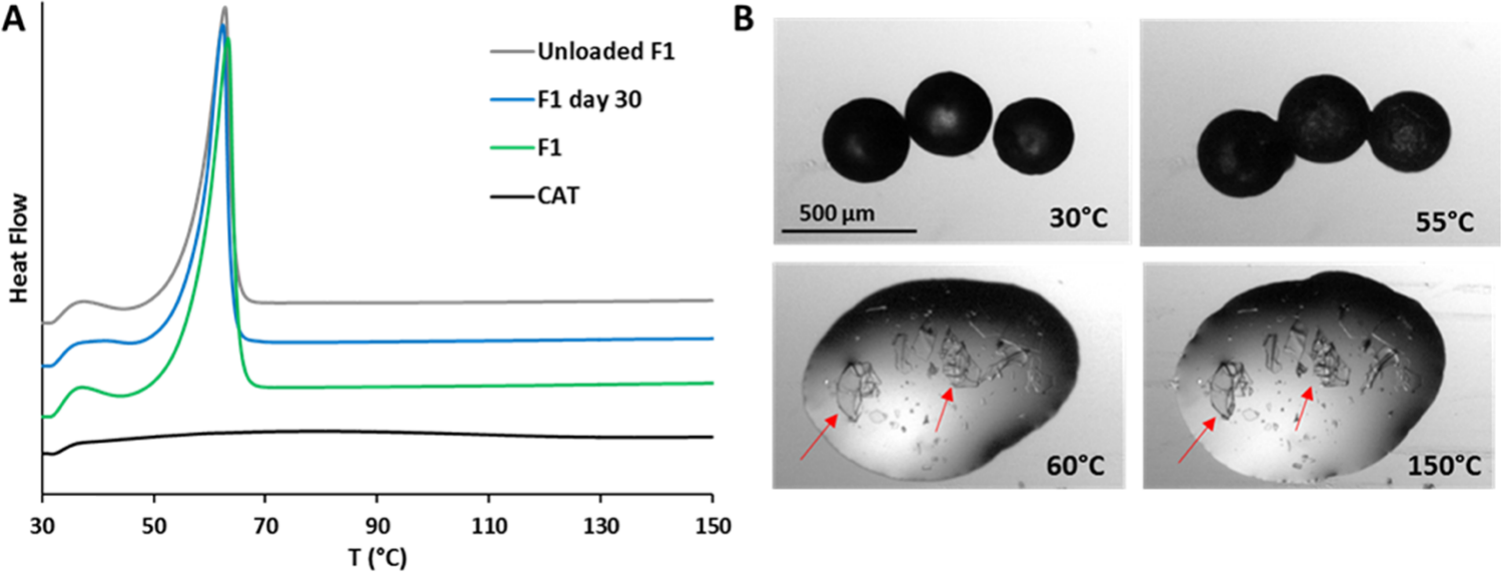
DSC analysis of CAT, unloaded F1, and CAT-loaded F1 immediately after preparation and after 1 month of storage (A) and hot stage microscopy (HSM) images of F1 SLMs (B).

Figure 2A shows the DSC analysis of the standard enzyme and SLMs. In the DSC analysis of CAT, only a broad weak endothermic peak ranging from about 30 to 130 °C was detected. This band, whose area depends on the amount of residual water in the sample, is due to water removal and is typical of lyophilized (amorphous) proteins at the solid state.^36,37^ In confirmation of this, the endothermic peak disappeared in a second heating scan of the same sample of CAT, as shown in Figure S3, SI. The thermogram of unloaded F1 SLMs showed an endothermic peak at 62.7 °C, related to the melting of Dynasan 114. As expected, in the case of CAT-loaded SLMs, a DSC profile similar to that of unloaded F1 was observed. Additionally, the DSC analysis of F1 after 1 month of storage showed no change in the thermal profile. These data suggest the absence of interactions between CAT and the lipid excipient and no alteration in the thermal properties of CAT-loaded SLMs. The DSC profiles of formulations F2–F4 (Figure S4, SI) also indicated the absence of carrier CAT interactions. The same heating process was performed on F1 SLMs while changes in the sample were investigated by hot stage microscopy (Figure 2B). In accordance with the DSC results, the melting of F1 SLMs started at about 55 °C and was complete at 60 °C. After melting of the lipid carrier, some solid CAT particles were observed in the melted excipient, as indicated by red arrows. The appearance of CAT particles was constant during the analysis with no change in their shape or color up to 150 °C.

### Integrity of CAT at the Solid State

The analysis of protein integrity was initially performed on CAT-loaded SLMs at the solid state because CAT remained in that physical state during the manufacturing process and after production of SLMs. Raman microspectroscopy has been widely used to monitor structural changes on proteins^38^ and it is particularly suitable for our purpose, since it can provide information about the secondary structure of a protein by direct analysis on the microparticles.^8^ The Raman spectra of standard CAT, CAT-unloaded, and CAT-loaded F1 SLMs are reported in Figure S5, SI. The spectrum of unloaded particles showed bands that could be assigned to the principal vibrations of the lipid carrier.^8,39^ The ester carbonyl stretching gave a weak signal around 1740 cm^–1^. The band at ca. 1460 cm^–1^, separated in two peaks, was characteristic of methyl vibrations at 1465 cm^–1^ and methylene scissoring vibrations at 1444 cm^–1^. The strong band at 1299 cm^–1^ corresponded to the C–C skeleton structure, whereas symmetric and asymmetric C–C stretching vibrations of the hydrophobic chains of the lipid were identified in the range between 1060 and 1130 cm^–1^. Finally, the multiple bands in the region 2850–2890 cm^–1^ are related to the various C–H stretching modes. All the characteristic bands of the lipid could be found unmodified in the Raman spectrum of CAT-loaded particles. The free enzyme gave a broad band around 1250–1400 cm^–1^, and the same signal could be found in the spectrum of CAT-loaded F1 SLMs (red arrows). However, very little information on protein integrity could be gathered by comparing the spectra of unloaded and CAT-loaded particles, as no marked difference between the two samples were detected. Unfortunately, the presence of CAT was associated with an intensive fluorescence interference. This represents a severe problem for Raman analysis, as even a weak fluorescence emission is often much stronger than the Raman scattering, resulting in a large background.^40^ Since fluorescence might interfere with Raman spectroscopy, but not with FT-IR spectroscopy, this method was therefore employed for the analysis of F1 SLMs. Figure S6, SI reports the FT-IR spectra in the region 900–2000 cm^–1^, where the typical amide stretching bands of the protein can be identified.^41^ The spectrum of standard CAT shows the amide I vibration, mainly related to the C=O stretching vibration, and the amide II vibration, which is the out-of-phase combination of the N–H in plane bend, and the C–N stretching vibration, at 1651 cm^–1^ and at 1541 cm^–1^, respectively. However, the low intensity of such signals compared to that of the strongest signals of the lipid carrier prevented a clear detection of the protein bands in the spectrum of CAT-loaded SLMs. Additionally, the small amount of loaded CAT (5% w/w) in F1 also negatively contributed to the correct detection of CAT bands in the sample. Even the analysis of F5, where the amount of encapsulated protein was 20% w/w, showed only a minor signal related to the presence of the protein (red arrows).

Therefore, the analysis of CAT-loaded SLMs at the solid state suggested the absence of interactions or incompatibilities between the drug and the carrier; however, it did not provide useful information on CAT integrity.

### Integrity of CAT Released from SLMs

The structural integrity of the enzyme was thus studied after release from the SLMs in the intestinal simulated medium, which was first tested to ensure it did not cause CAT denaturation. To this purpose, both the secondary and tertiary structures of the encapsulated protein were examined.

**Figure 3:**
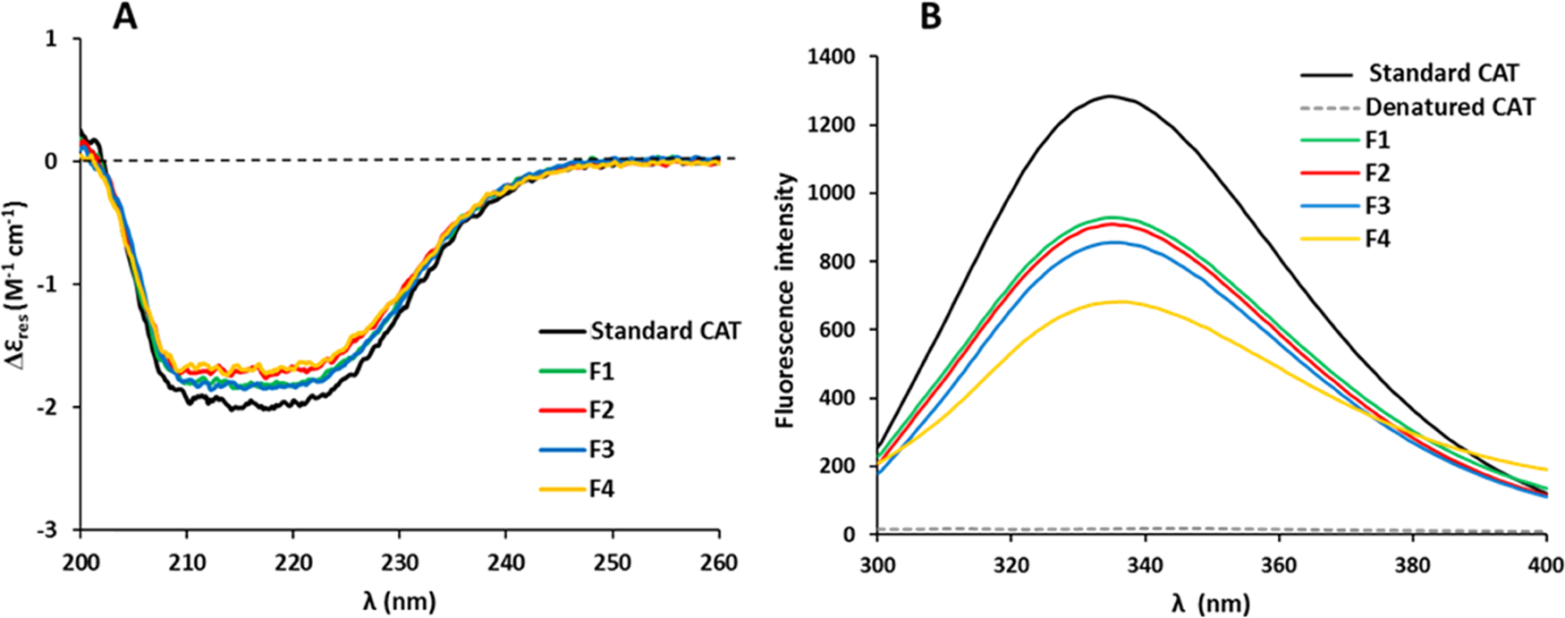
Secondary structure analysis of standard CAT and encapsulated CAT released from SLMs (F1–F4) by CD spectroscopy (A) and conformational analysis of encapsulated CAT released from SLMs by fluorescence spectroscopy (B).

CD spectroscopy is the technique of choice to investigate the secondary structure of proteins in solution, thanks to its sensitivity to the conformational arrangement of peptide bonds in the protein backbone.^42^ The far-UV CD spectra of CAT samples released from SLMs (Figure 3A) showed some differences in terms of intensity: a possible explanation of this behavior might be that F2- and F4-released CAT samples are relatively more affected than F1- and F3-samples by the spray congealing process and their encapsulation within SLMs. However, the overall secondary structure of CAT is not dramatically perturbed by the encapsulation and release processes, as the CD profile of SLM-released CAT is substantially similar to that of standard CAT.

Due to the inherent fluorescence of aromatic amino acids like tyrosine, tryptophan, and phenylalanine, fluorescence emission spectra can be used to monitor the stability of the protein tertiary structure.^43^ When excited at 280 nm, proteins generate an emission band, which is generally located between 300 and 350 nm and is dependent on the local environment and/or the polarity of the solvent.^44^ The fluorescence spectrum of standard CAT was compared with those of CAT released from SLMs with different compositions (Figure 3B). The fluorescence spectrum of standard CAT showed an emission band with a maximum intensity at 334.5 nm, in accordance with data from the literature.^45,46^ In contrast, the negative control (denatured CAT) showed a weak fluorescence emission characterized by the absence of the band at 334 nm. Compared to the positive control, solutions of CAT released from the particles exhibited emission peaks with decreased intensity but a similar band shape, indicating that the tertiary structure of the protein was mainly preserved. Only minor changes in the band maxima were observed and specifically they were located at 335.0, 336.0, 335.5, and 337.0 nm for F1, F2, F3, and F4, respectively. Changes in the fluorescence emission band may imply that the microenvironment of the aromatic residues in the enzyme was altered after the encapsulation in SLMs. The influence of the excipients on the tertiary structure of the encapsulated protein has been reported before. For example, fluorescence spectra of BSA incorporated in poly(lactic-*co*-glycolic acid) (PLGA)-based microspheres showed a change toward lower wavelengths (blue shift), indicating higher compactness, in the case of formulations containing poly(ethylene glycol) (PEG) as the stabilizer.^47^ It was hypothesized that effects of steric interactions as well as energetic stabilization were the reasons for the change in the protein tertiary structure. In particular, the hydrophobic components of the excipient led to a tighter packing of the protein molecules, present either on the surface or inside the microsphere. Accordingly, the hydrophobic chains of the glycerides used as the carrier of SLMs may have determined a slightly different confinement of CAT within the lipid matrix.

### CAT Activity

It is generally recognized that the activity of an enzyme is strongly dependent on its conformational integrity. In the case of tetrameric proteins such as CAT, protein dissociation into subunits, protein unfolding, and protein denaturation are the most common events that can lead to a loss of catalytic activity. For example, dissociation of CAT into subunits has been observed at pH extremes,^48,49^ in the presence of denaturants such as sodium *n*-dodecyl sulfate,^50^ and after lyophilization.^51^ In all these cases, the enzymatic activity of CAT was compromised. However, for metal-containing enzymes and multi-subunit enzymes, such as CAT, the inactivation of the enzyme may occur without detectable conformational changes in the macromolecule.^49^ Therefore, enzymes are useful model proteins as the retention of their native structure can be evaluated indirectly by monitoring their catalytic activity using simple assays. Nevertheless, it is also possible that small perturbations in the protein structure do not affect the catalytic activity. For example, Prakash et al.^52^ observed that standard CAT can dissociate into enzymatically active folded dimers in the presence of low amounts of specific denaturants. The dimer showed a slightly higher enzymatic activity (although with altered structural properties) compared to the native tetramer. Therefore, regardless of the conformational integrity studies, the measurement of CAT catalytic activity is fundamental to understand the stability of the protein after the SC process.

**Figure 4:**
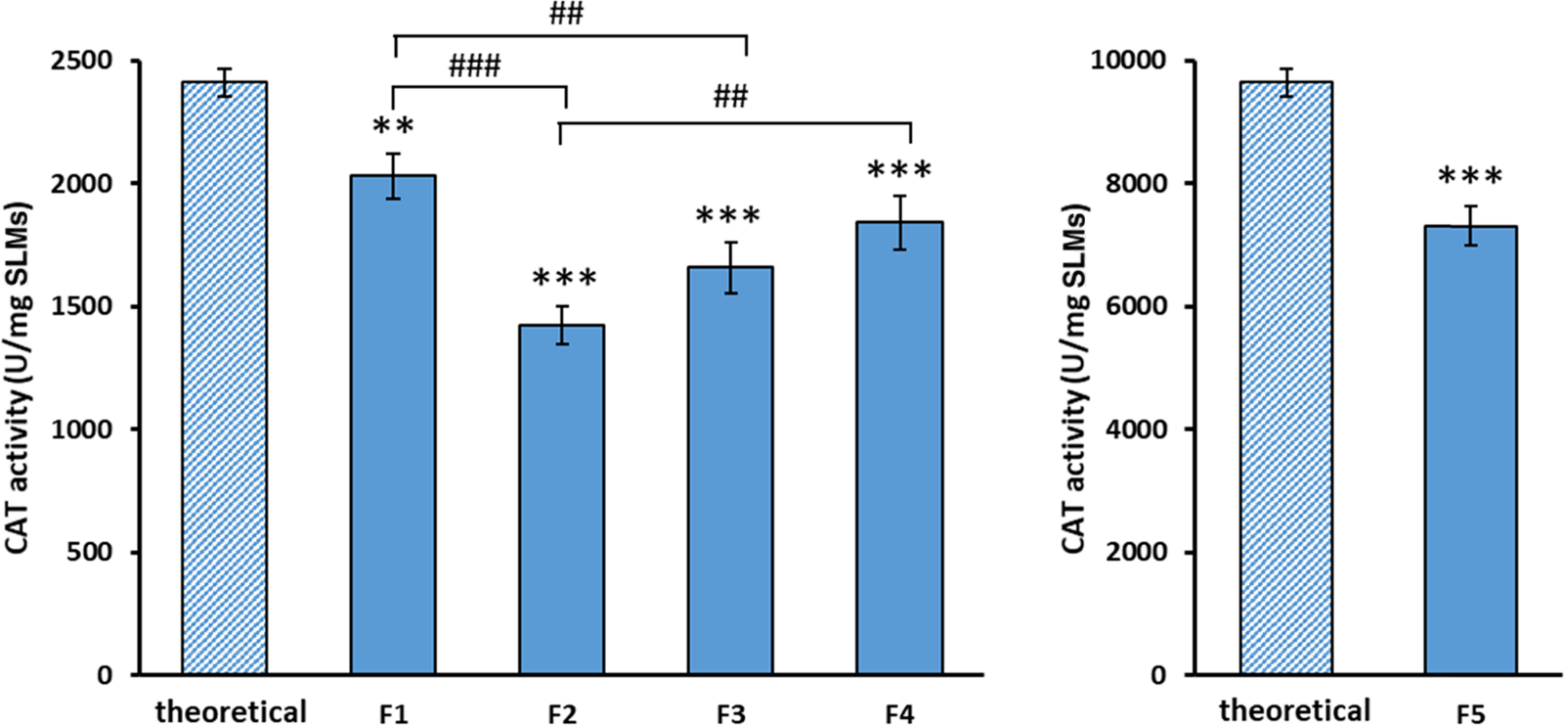
Activity of encapsulated CAT in SLMs. Theoretical CAT activity of SLMs calculated from free CAT activity and CAT drug loadings is also reported. Values are expressed as mean (*n* = 3) ± S.D. **p < 0.01 and ***p < 0.001, significant difference compared to the theoretical activity. ^##^*p* < 0.01 and ^###^*p* < 0.001, significant difference between the indicated groups.

Considering the activity of free standard CAT, the SLMs with 5% w/w drug should present a CAT activity, defined as theoretical activity, of ca. 2400 U/mg. First, the stability of CAT after encapsulation was studied by measuring the enzyme activity in SLMs immediately after production. The results, shown in Figure 4, indicated that CAT activity after encapsulation varied in the different formulations, decreasing in the order F1 > F4 > F3 > F2. These differences can be attributed to the temperature employed in the SC process, which depends on the melting temperature of the lipid carrier: Dynasan 114 alone (F1) has a melting temperature of 55–58 °C. In the formulations containing Dynasan 114 combined with another lipid carrier, the melting temperature of the lipid binary mixture is influenced by the melting temperatures of the other stearic-based glycerides. Specifically, the melting temperatures of these materials increase by increasing the number of fatty acid chains of the glyceride. Hence, the melting temperature of glyceryl monostearate, distearate, and tristearate are 58–59, 61, and 73 °C, respectively. Therefore, the loss of CAT activity after encapsulation changed in accordance with the temperature used in the process. However, it should be noted that this thermal degradation was much more limited compared to the activity loss observed by heating CAT solutions. In the latter, a significant denaturation was observed already at 40 °C and it was complete at 60 °C (see Figure S2, SI). Indeed, it should be considered that a protein in its solid form is generally much more stable than in solution.^53^ In fact, solid lyophilized proteins are usually in an amorphous glassy form, in which the protein local motion is restricted, hence, the rates of many chemical degradation reactions are reduced.^54,55^

To exclude that differences in activities were related to a different amount of loaded protein and confirm that residual CAT activity depended upon the process temperature, the amount of protein encapsulated in the SLMs was determined. As shown in Table 2, the amount of encapsulated protein was similar for the four formulations at 5% w/w loading, i.e., F1–F4 SLMs, while it was correspondingly higher for F5, having a theoretical CAT loading of 20% w/w. These results evidently confirmed that CAT activity mainly changed in accordance with the temperature used in the process and was not related to the total amount of loaded protein. Moreover, the similar protein content for formulation with equal theoretical drug loadings indicated that the SC process allowed protein encapsulation of SLMs independently on lipid composition and process temperature. However, slightly lower process yields (Table 2) were observed for lower-temperature process formulations (e.g., F1 and F5).

**Table 2:** Protein Content, CAT Activity, and Process Yield of CAT-Loaded SLMs

sample	protein content (μg/mg SLMs)	CAT activity (U/mg SLMs)	yield (%)
F1	64.3 ± 1.7	2033 ± 92	64.5
F2	61.8 ± 1.0	1423 ± 78	82.0
F3	65.4 ± 1.2	1659 ± 103	74.0
F4	64.7 ± 0.6	1841 ± 108	70.4
F5	241.9 ± 1.4	7311 ± 321	67.3

One question could arise regarding the influence of drug loading in the retention of CAT activity after encapsulation. Is the residual activity after the SC process independent of the amount of CAT loaded in the formulation, or is it proportional to it? To this regard, the activity of formulation F5, loaded with 20% w/w CAT, was evaluated. As shown in Figure 4, compared to a theoretical activity of ca. 9600 U/mg SLMs, the activity of F5 SLMs corresponded to 7311 U/mg. Therefore, considering the retention of activity in relation to the specific drug loadings, the residual CAT activity after its processing was similar, i.e., 84 and 76% for F1 and F5, respectively (*p* < 0.01).

From these results, we can conclude that SC allowed CAT encapsulation with consistent protein loading values and good yield. As hypothesized, if the biological drug is loaded at the solid state, the risk of denaturation during the formulation process is markedly reduced. Even by using relatively high working temperatures, higher than 60–70 °C, most biological activity was retained. Differently from the formulation variables, the drug loading had a minor influence on the retention of activity after encapsulation by SC.

### CAT Protection from Gastric Degradation and In Vitro Release Study

With the aim of evaluating the ability of the SLMs in protecting CAT from gastric degradation, the main features of the gastric environment should be considered. The gastric pH varies from 1.0–1.5 (basal fasting conditions) to 5–7 after meal ingestion, depending on the type of meal and its buffering capacity.^25^ As CAT was found inactive at pH ≤ 2 (Figure 1), the most unfavorable conditions were simulated by selecting an extremely acidic pH (pH 1.2). In addition to the acidic pH, also the gastric digestive hydrolases play a major role in determining the integrity of orally administered biotherapeutics. Thus, both the effect of extremely acidic pH (pH 1.2, consistent with fasting conditions) as well as the effect of the main gastric hydrolases, pepsin and gastric lipase, at a pH of 4.5, representing the fed stomach conditions were considered.

**Figure 5:**
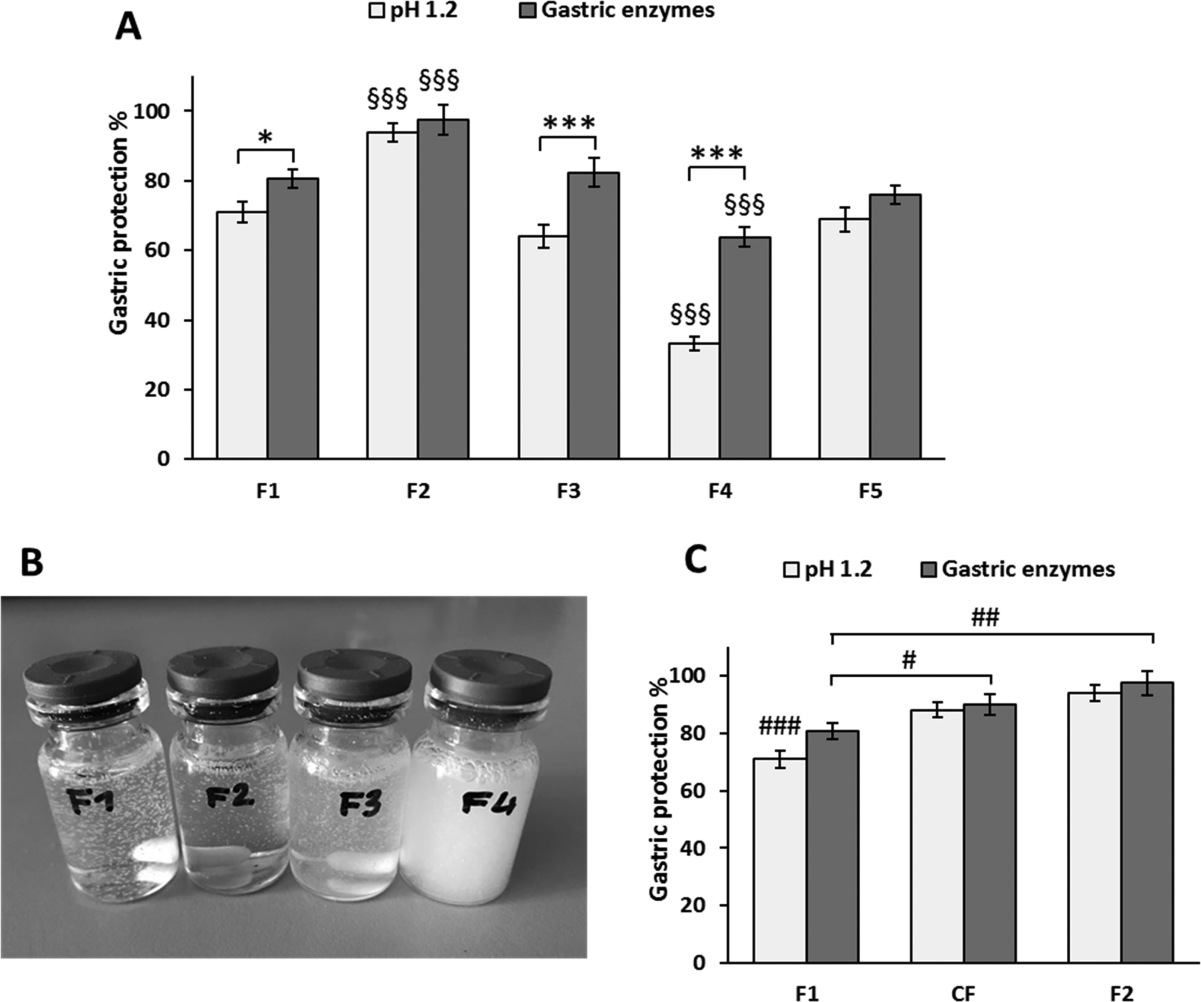
Estimated gastric protection ability of SLMs calculated from the residual CAT activity after 1 h incubation in media simulating gastric conditions (A), appearance of F1, F2, F3, and F4 SLM suspensions after 1 h of incubation in gastric medium of pH 1.2 (B) and effect of different chain lengths of triglycerides on the gastric protection ability of SLMs (C). *p < 0.05 and ***p < 0.001, significant difference between residual CAT activities after treatment with acidic pH and gastric enzymes (same formulation). ^§§§^*p* < 0.001, significant difference compared to all other SLMs after incubation in the same conditions. ^###^*p* < 0.001, significant difference compared to CF and F2 SLMs after incubation in a pH of 1.2. ^#^*p* < 0.05, ^##^*p* < 0.01, significant difference between the indicated groups after incubation with gastric enzymes.

Figure 5A shows the gastric protection exerted by the SLMs, considering the 100% as complete protection of the encapsulated protein from denaturation in the simulated gastric medium. After treatment with acidic pH, the formulation based entirely on Dynasan 114 (F1) preserved about 70% of the enzymatic activity of CAT. Modifications in the lipid composition led to different results: the addition of glyceryl tri- (F2), di- (F3), and mono- (F4) stearate led to 95, 65, and 35% of protection, respectively. SLMs with increased drug loading (F5) did not show significant differences from the corresponding formulation with a lower drug amount (*p* > 0.05). The dispersions of the SLMs in the gastric medium at pH 1.2 (Figure 5B) were substantially different in their appearance: a very clear transparent suspension was observed in the case of F2, whereas a more opalescent suspension was obtained upon dispersions of F1 and F3, indicating a modest emulsification and solubilization of the carrier. The SLMs of formulation F4 were the least efficient for the gastric protection of CAT: the SLMs did not maintain their integrity, as after 1 h of incubation the suspension became opaque because of the high amount of lipid released in the medium.

The protection of SLMs from CAT inactivation in the presence of gastric digestive enzymes showed the same trend observed for acidic pH (i.e., F2 > F1 ∼ F5 ∼ F3 > F4). However, the effect of digestive enzymes on CAT activity was generally lower. Whereas for F2 and F5 no significant difference between CAT residual activity in the two conditions was observed (*p* > 0.05), the effect of acidic pH on CAT activity was significantly higher (*p* < 0.001) than that caused by the digestive enzymes for F3 and F4 SLMs. This could be ascribed to the different mechanism of CAT inactivation: acidic conditions caused almost complete CAT inactivation already at pH 2 (Figure S2C, SI), hence, probably protein particles exposed to the medium (e.g., CAT particles at the surface of SLMs) were immediately inactivated. Differently, the inactivation by pepsin conceivably is an enzymatic reaction, which occurs in solution and involves the fraction of CAT released from SLMs. Furthermore, proteolytic digestion of CAT by pepsin is a slower process in which peptide bonds in specific protein regions (aromatic amino acids from the N-terminus of proteins) are cleaved.^56^ As these processes need a longer time, the extent of loss of CAT activity was limited compared to acidic pH conditions.

To explain the different protection ability of SLMs, hydrophobicity of the lipids should be considered. This property depends both on the chain length and the substitution degree (mono-, di-, or tri-) of the glyceride. From the obtained results, it appeared that longer chain lengths increased the protection ability (i.e., F2 showed higher CAT residual activity compared to all other SLMs, *p* < 0.001). To confirm the effect of the glyceride chain length on gastric protection, an additional formulation was produced. This control formulation, named CF, is composed of Dynasan 114 (C14) and Dynasan 116 (C16) in a 1:1 weight ratio, and thus it is “intermediate” between F1 formulation (only Dynasan 114, C14) and F2 (Dynasan 114, C14, and Dynasan 118, C18). The results (Figure 5C) show an intermediate protection compared to F1 and F2 SLMs, confirming the hypothesis that longer chain fatty acids increased the residual catalytic activity. Apparently, the protection of the labile protein was strictly related to lipid wettability, which decreased proportionally with the fatty acid chains of the lipid.^57^ Moreover, the obtained results suggested that the protection ability decreased by using mono- and diglycerides. Accordingly, partial substituted glycerides are considered “surface active” in nature as they have polar functional groups as well as nonpolar hydrocarbon chains^58^ and present hydrophilic–lipophilic balance (HLB) values of 2–5.^59^ Therefore, the obtained results showed that the protection ability of glyceride-based SLMs increased with higher hydrophobicity and lower polarity of the carrier.

**Figure 6:**
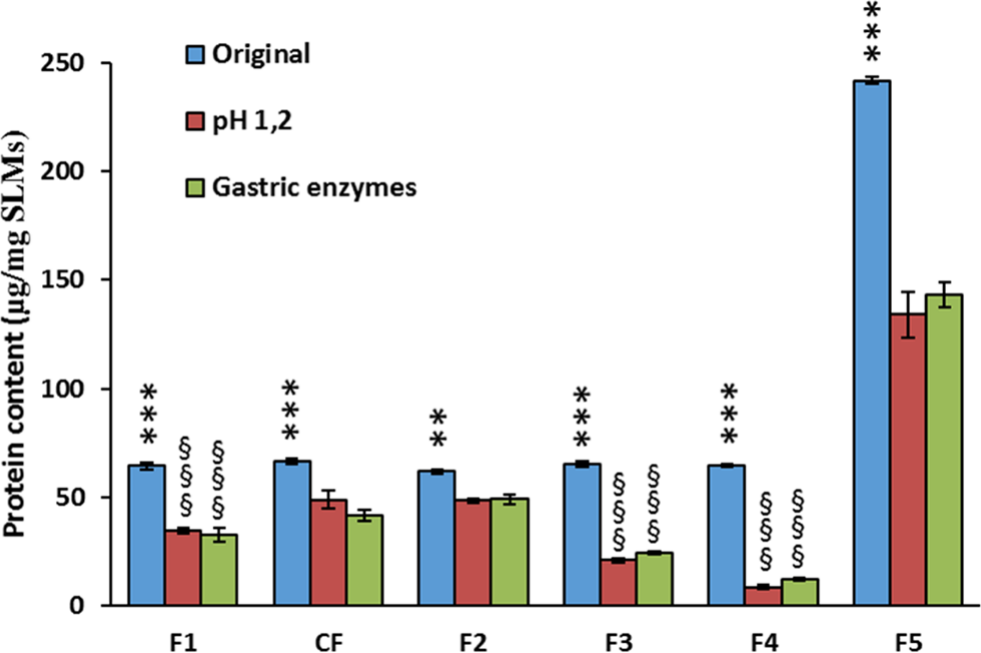
Total protein content in SLMs after production (original) and after 1 h of incubation in media simulating gastric conditions. **p < 0.01 and ***p < 0.001, significant difference between original protein contents and protein contents after treatment with acidic pH and gastric enzymes (same formulation). ^§§§^*p* < 0.001, significant difference compared to all other SLMs after incubation in the same conditions.

The protein content of SLMs after incubation in simulated gastric conditions is shown in Figure 6. In all formulations, the protein content decreased after gastric passage, as the protein was partially released in the simulated gastric fluid. The extent of the protein content loss for the different SLMs was consistent with the loss of CAT activity (e.g., F2 showed the highest retention of protein amounts as well as the highest protection of enzymatic activity), suggesting that CAT inactivation was mainly related to protein release from the SLMs during the incubation time. Additionally, the treatments with acidic pH and with digestive enzymes resulted in similar residual protein contents (*p* > 0.05), supporting the hypothesis that the higher CAT activity loss by acidic pH was not caused by an enhanced protein release from the SLMs, but rather depended on the stronger denaturating effect on CAT particles exposed to the acidic medium.

In our previous study,^10^ Dynasan 114-based SLMs showed good ability to protect the encapsulated protein from gastric inactivation and SLMs with diameters between 150 and 250 μm resulted in a higher protection, compared to SLMs with the 50–150 μm size. As the protection ability was proportional to the particle size, it was hypothesized that the protein located on the external surface of the particle, or closer to it, was inactivated by the direct contact with the gastric fluid, whereas the drug encapsulated in the inner part was efficiently protected. Thus, a multiparticulate system with a larger specific surface area would have less potential for gastric protection. Besides the particle size, the composition is also supposed to have a profound influence on the gastrointestinal stability. This study showed that the protection from gastric inactivation of SLMs can be modulated by using combinations of different glycerides. Even considering that SLMs are matrix systems (with the drug evenly distributed within the carrier) where the protein on the surface is inevitably inactivated, by selecting SLMs with suitable composition, a good protection (up to 90%) can be achieved. The lipid composition of SLMs, therefore, is fundamental to provide an adequate protection. To conclude, in the selection of lipid excipients for oral delivery of biological compounds, the protection from degradation in the stomach can be enhanced by: (i) increasing the fatty acid chain length of the glyceride and (ii) increasing the degree of substitution of the glyceride.

**Figure 7:**
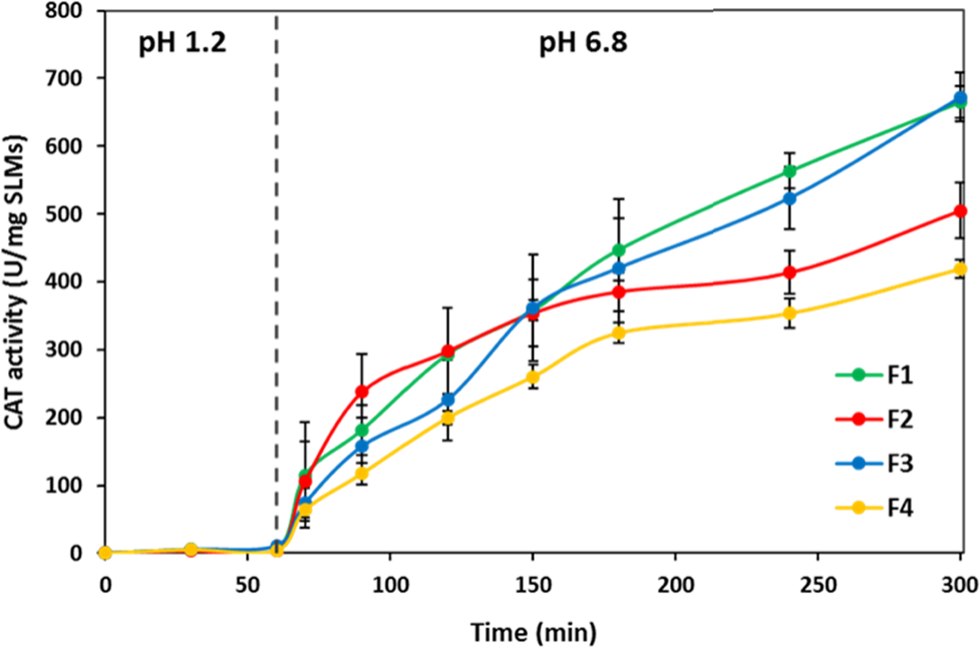
In vitro release of CAT from F1, F2, F3, and F4 SLMs in a simulated gastric fluid (HCl solution, pH = 1.2) for 1 h, followed by 4 h at pH 6.8 to simulate the SLM transit in the intestine.

In vitro release study was performed using two different media to simulate the transit of the SLMs through the GIT (Figure 7). As expected, almost no CAT activity was observed at pH 1.2. The appearance of CAT activity upon pH increase to 6.8 was immediate for all formulations, confirming the ability of SLMs to provide sufficient protection from the harsh gastric pH and, at the same time, to allow protein release in the intestine. The release profiles of F1 and F3 SLMs were similar and showed a gradual increase over 4 h giving the highest value of CAT activity (almost 700 U/mg SLMs). F2 SLMs showed a fast CAT release immediately after the pH switch, followed by a more controlled release of CAT, caused by the slow erosion of the lipid matrix containing triglycerides with longer hydrophobic chains compared to F1. Finally, F4 SLMs showed the lowest CAT activity (504 U/mg SLMs after 4 h), probably due to the poor efficiency of this formulation for gastric protection, resulting in the loss of most CAT activity during gastric transit.

### Effect of SLMs on Cell Viability

**Figure 8:**
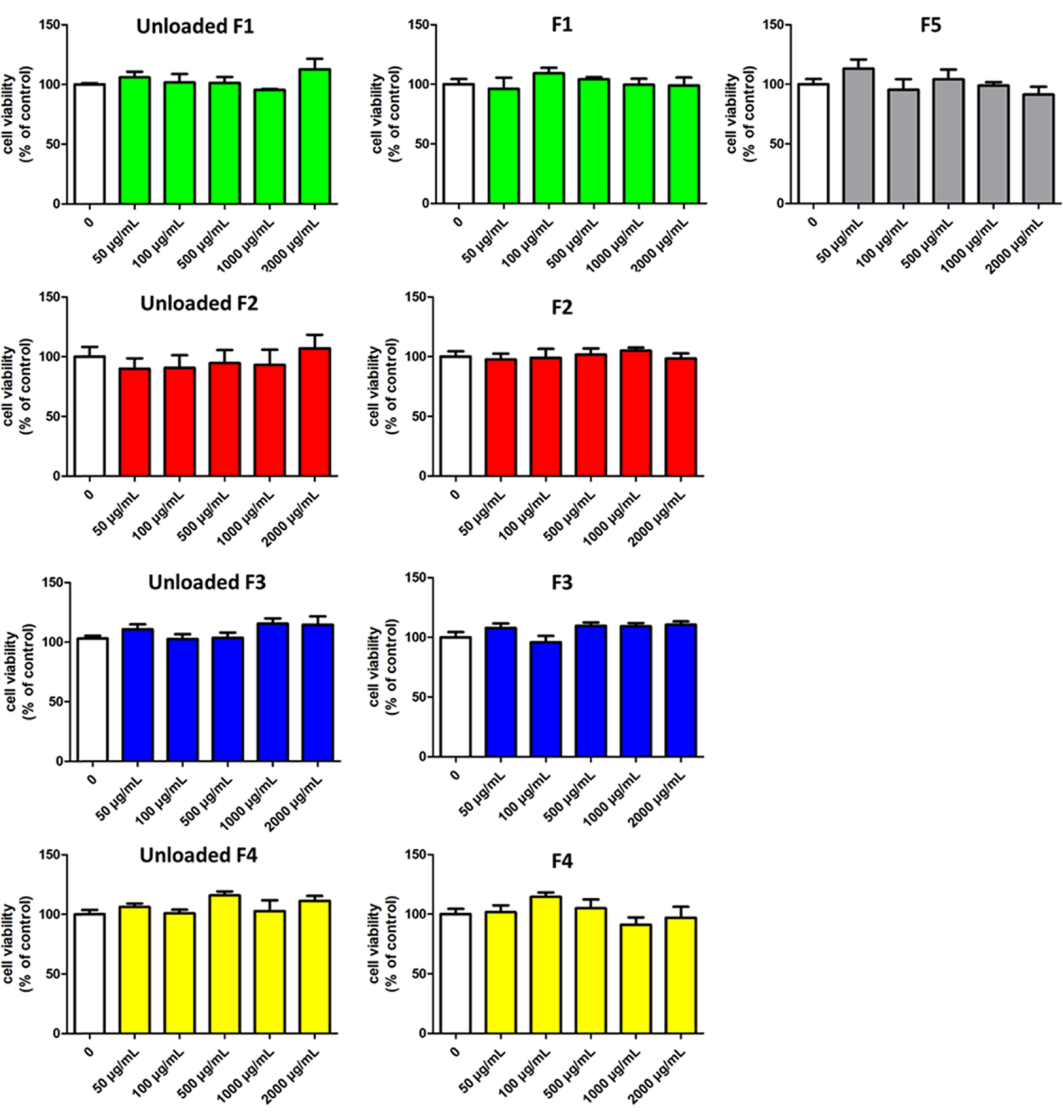
Cell viability of HT29 after 24 h incubation of CAT-loaded SLMs and unloaded SLMs at increasing concentrations (50–2000 μg/mL). Values are expressed as mean (*n* = 3) ± SD. *p* > 0.05, therefore not statistically significant according to the one-way analysis of variance (ANOVA) followed by the Bonferroni post hoc test (GraphPad Prism, GraphPad software Inc., CA).

Lack of toxicity and biocompatibility are fundamental requisites of oral delivery carriers. To assess the absence of cytotoxicity of the designed systems, the effect of SLMs upon exposure to intestinal cells was evaluated for both drug-free (unloaded) formulations and CAT-loaded SLMs on the human colon adenocarcinoma intestinal cell line (HT29) by means of the MTT assay. Data are reported in Figure 8. After 24 h of incubation, SLMs at concentrations up to 2000 μg/mL were completely safe on HT29 cells, as indicated by viability values not significantly different from the control (*p* > 0.05 compared to the control). No significant difference was observed between unloaded and CAT-loaded formulations. Indeed, all SLM formulations showed excellent intestinal biocompatibility.

## Conclusions

In the development of oral biotherapeutics, protein biological activity must be maintained during the preparation, storage, and after administration. Catalase (CAT) was encapsulated in SLMs based on different long-chain glycerides. Despite the employment of a thermolabile protein, the spray congealing technology allowed CAT encapsulation with consistent protein-loading values, good yield, and preservation of most of its biological activity, due to the loading of the drug at the solid state. Circular dichroism and fluorescence spectroscopy confirmed that both the secondary and tertiary structures were mostly retained. Depending on the carrier employed, the protection of CAT from gastric conditions (acidic pH and digestive enzymes) achieved by the SLMs ranged from 35 to 95%. Specifically, the residual catalytic activity was higher with the increasing of the fatty acid chain length and the increasing of the degree of substitution of the glyceride. Whereas the SLM particle size and the carrier selection were of fundamental importance, the drug-loading degree did not influence either the protein integrity or the protection from the gastric environment. All the examined formulations showed excellent intestinal biocompatibility. Overall, SLMs based entirely on glyceryl trimyristate (F1) showed a good compromise between the retention of CAT activity during the process and its protection from simulated gastric conditions, as well as the protein release in the intestinal environment. In conclusion, this work provides new insights into the relevant properties to be considered when SLMs are designed for the oral delivery of biotherapeutics, as well as an understanding of the influence of these properties on the protein stability and activity.

## Supporting Information Available

Yellow complex between H_2_O_2_ and ammonium molybdate; free CAT characterization: kinetic analysis, effect of pH and temperature on CAT activity; DSC analysis of free CAT performed between 30 and 250 °C in two following heating scans; additional DSC analysis of F2, F3, and F4 SLMs; Raman spectra of CAT, unloaded F1 SLMs, and CAT-loaded F1 SLMs; FT-IR spectra of CAT, unloaded F1 SLMs, and F1 SLMs loaded with CAT at 5% (F1) and 20% (F5); references (PDF)

## References

[ref1] Koetting M. C.; Guido J. F.; Gupta M.; Zhang A.; Peppas N. A. PH-Responsive and Enzymatically-Responsive Hydrogel Microparticles for the Oral Delivery of Therapeutic Proteins: Effects of Protein Size, Crosslinking Density, and Hydrogel Degradation on Protein Delivery. J. Controlled Release 2016, 221, 18–25. 10.1016/j.jconrel.2015.11.023. PMC4734382 26616761

[ref2] Date A. A.; Hanes J.; Ensign L. M. Nanoparticles for Oral Delivery: Design, Evaluation and State-of-the-Art. J. Controlled Release 2016, 240, 504–526. 10.1016/j.jconrel.2016.06.016. PMC5064878 27292178

[ref3] Frokjaer S.; Otzen D. E. Protein Drug Stability: A Formulation Challenge. Nat. Rev. Drug Discovery 2005, 4, 298–306. 10.1038/nrd1695. 15803194

[ref4] Mcclements D. J. Encapsulation, Protection, and Delivery of Bioactive Proteins and Peptides Using Nanoparticle and Microparticle Systems: A Review. Adv. Colloid Interface Sci. 2018, 253, 1–22. 10.1016/j.cis.2018.02.002. 29478671

[ref5] Bertoni S.; Dolci L. S.; Albertini B.; Passerini N. Spray Congealing: A Versatile Technology for Advanced Drug Delivery Systems. Ther. Delivery 2018, 9, 833–845. 10.4155/tde-2018-0049. 30444462

[ref6] Maschke A.; Becker C.; Eyrich D.; Kiermaier J.; Blunk T.; Göpferich A. Development of a Spray Congealing Process for the Preparation of Insulin-Loaded Lipid Microparticles and Characterization Thereof. Eur. J. Pharm. Biopharm. 2007, 65, 175–187. 10.1016/j.ejpb.2006.08.008. 17070025

[ref7] Zaky A.; Elbakry A.; Ehmer A.; Breunig M.; Goepferich A. The Mechanism of Protein Release from Triglyceride Microspheres. J. Controlled Release 2010, 147, 202–210. 10.1016/j.jconrel.2010.07.110. 20659511

[ref8] Di Sabatino M.; Albertini B.; Kett V. L.; Passerini N. Spray Congealed Lipid Microparticles with High Protein Loading: Preparation and Solid State Characterisation. Eur. J. Pharm. Sci. 2012, 46, 346–356. 10.1016/j.ejps.2012.02.021. 22465062

[ref9] Rosiaux Y.; Jannin V.; Hughes S.; Marchaud D. Solid Lipid Excipients — Matrix Agents for Sustained Drug Delivery. J. Controlled Release 2014, 188, 18–30. 10.1016/j.jconrel.2014.06.004. 24929038

[ref10] Bertoni S.; Albertini B.; Dolci L. S.; Passerini N. Spray Congealed Lipid Microparticles for the Local Delivery of β -Galactosidase to the Small Intestine. Eur. J. Pharm. Biopharm. 2018, 132, 1–10. 10.1016/j.ejpb.2018.08.014. 30176285

[ref11] Christophersen P. C.; Zhang L.; Yang M.; Nielsen H. M.; Müllertz A.; Mu H. Solid Lipid Particles for Oral Delivery of Peptide and Protein Drugs I – Elucidating the Release Mechanism of Lysozyme during Lipolysis. Eur. J. Pharm. Biopharm. 2013, 85, 473–480. 10.1016/j.ejpb.2013.07.017. 23911434

[ref12] Christophersen P. C.; Zhang L.; Müllertz A.; Nielsen H. M.; Yang M.; Mu H. Solid Lipid Particles for Oral Delivery of Peptide and Protein Drugs II - The Digestion of Trilaurin Protects Desmopressin from Proteolytic Degradation. Pharm. Res. 2014, 31, 2420–2428. 10.1007/s11095-014-1337-z. 24623481

[ref13] Christophersen P. C.; Vaghela D.; Müllertz A.; Yang M.; Nielsen H. M.; Mu H. Solid Lipid Particles for Oral Delivery of Peptide and Protein Drugs III — the Effect of Fed State Conditions on the In Vitro Release and Degradation of Desmopressin. AAPS J. 2014, 16, 875–883. 10.1208/s12248-014-9619-2. 24875052 PMC4070260

[ref14] Ma Z.; Lim T. M.; Lim L.-Y. Pharmacological Activity of Peroral Chitosan–insulin Nanoparticles in Diabetic Rats. Int. J. Pharm. 2005, 293, 271–280. 10.1016/j.ijpharm.2004.12.025. 15778065

[ref15] Soudry-Kochavi L.; Naraykin N.; Nassar T.; Benita S. Improved Oral Absorption of Exenatide Using an Original Nanoencapsulation and Microencapsulation Approach. J. Controlled Release 2015, 217, 202–210. 10.1016/j.jconrel.2015.09.012. 26381898

[ref16] Gracia R.; Yus C.; Abian O.; Mendoza G.; Irusta S.; Sebastian V.; Andreu V.; Arruebo M. Enzyme Structure and Function Protection from Gastrointestinal Degradation Using Enteric Coatings. Int. J. Biol. Macromol. 2018, 119, 413–422. 10.1016/j.ijbiomac.2018.07.143. 30048728

[ref17] Niu Z.; Conejos-sánchez I.; Grif B. T.; Driscoll C. M. O.; Alonso M. J. Lipid-Based Nanocarriers for Oral Peptide Delivery. Adv. Drug Delivery Rev. 2016, 106, 337–354. 10.1016/j.addr.2016.04.001. 27080735

[ref18] Chen C.; Fan T.; Jin Y.; Zhou Z.; Yang Y.; Zhu X.; Zhang Z.; Zhang Q.; Huang Y. Orally Delivered Salmon Calcitonin-Loaded Solid Lipid Nanoparticles Prepared by Micelle–double Emulsion Method via the Combined Use of Different Solid Lipids. Nanomedicine 2013, 8, 1085–1100. 10.2217/nnm.12.141. 23075315

[ref19] Diakidou A.; Vertzoni M.; Abrahamsson B.; Dressman J.; Reppas C. Simulation of Gastric Lipolysis and Prediction of Felodipine Release From a Matrix Tablet in the Fed Stomach. Eur. J. Pharm. Sci. 2009, 37, 133–140. 10.1016/j.ejps.2009.02.003. 19429420

[ref20] Li Y.; Zhou Y.; Han W.; Shi M.; Zhao H.; Liu Y.; Zhang F.; Zhang J. Novel Lipidic and Bienzymatic Nanosomes for Efficient Delivery and Enhanced Bioactivity of Catalase. Int. J. Pharm. 2017, 532, 157–165. 10.1016/j.ijpharm.2017.09.006. 28888973

[ref21] Qi C.; Chen Y.; Jing Q.; Wang X. Preparation and Characterization of Catalase-Loaded Solid Lipid Nanoparticles Protecting Enzyme against Proteolysis. Int. J. Mol. Sci. 2011, 12, 4282–4293. 10.3390/ijms12074282. 21845078 PMC3155351

[ref22] He L.; Lan W.; Zhao Y.; Chen S.; Liu S.-L.; Cen L.; Cao S. W.; Dong L.; Jin R.; Liu Y. Characterization of biocompatible pig skin collagen and application of collagen-based films for enzyme immobilization. RSC Adv. 2020, 10, 7170–7180. 10.1039/C9RA10794K. PMC9049748 35493877

[ref23] Di Sabatino M.; Albertini B.; Kett V. L.; Passerini N. Spray congealed lipid microparticles with high protein loading: Preparation and solid state characterization. Eur. J. Pharm. Sci. 2012, 46, 346–356. 10.1016/j.ejps.2012.02.021. 22465062

[ref24] Sassene P. J.; Fanø M.; Mu H.; Rades T.; Aquistapace S.; Schmitt B.; Cruz-Hernandez C.; Wooster T. J.; Müllertz A. Comparison of lipases for in vitro models of gastric digestion: lipolysis using two infant formulas as model substrates. Food Funct. 2016, 7, 3989–3998. 10.1039/C6FO00158K. 27711870

[ref25] Sams L.; Paume J.; Giallo J.; Carrière F. Relevant pH and Lipase for in Vitro Models of Gastric Digestion. Food Funct. 2016, 7, 30–45. 10.1039/C5FO00930H. 26527368

[ref26] Ulleberg E. K.; Comi I.; Holm H.; Herud E. B.; Jacobsen M.; Vegarud G. E. Human Gastrointestinal Juices Intended for Use in In Vitro Digestion Models. Food Dig. 2011, 2, 52–61. 10.1007/s13228-011-0015-4. 22558059 PMC3339592

[ref27] Renukuntla J.; Vadlapudi A. D.; Patel A.; Boddu S. H. S.; Mitra A. K. Approaches for Enhancing Oral Bioavailability of Peptides and Proteins. Int. J. Pharm. 2013, 447, 75–93. 10.1016/j.ijpharm.2013.02.030. 23428883 PMC3680128

[ref28] Kaushal J.; Seema; Singh G.; Arya S. K. Immobilization of Catalase onto Chitosan and Chitosan–bentonite Complex: A Comparative Study. Biotechnol. Rep. 2018, 18, e00258 10.1016/j.btre.2018.e00258. PMC5989589 29876307

[ref29] Erol K.; Cebeci B. K.; Köse K.; Köse D. A. Effect of Immobilization on the Activity of Catalase Carried by Poly(HEMA-GMA) Cryogels. Int. J. Biol. Macromol. 2019, 123, 738–743. 10.1016/j.ijbiomac.2018.11.121. 30452980

[ref30] Parihar A. K. S.; Srivastava S.; Patel S.; Singh M. R.; Singh D. Novel Catalase Loaded Nanocores for the Treatment of Inflammatory Bowel Diseases. Artif. Cells, Nanomed., Biotechnol. 2017, 45, 981–989. 10.1080/21691401.2016.1198363. 27322626

[ref31] Abdel-mageed H. M.; Fahmy A. S.; Shaker D. S.; Saleh A. Development of Novel Delivery System for Nanoencapsulation of Catalase: Formulation, Characterization, and in Vivo Evaluation Using Oxidative Skin Injury Model. Artif. Cells, Nanomed., Biotechnol. 2018, 46, 362–371. 10.1080/21691401.2018.1425213. 29336165

[ref32] Scalia S.; Young P. M.; Traini D. Solid lipid microparticles as an approach to drug delivery. Expert Opin. Drug Delivery 2015, 12, 583–599. 10.1517/17425247.2015.980812. 25391992

[ref33] Chen W.; Palazzo A.; Hennink W. E.; Kok R. J. Effect of Particle Size on Drug Loading and Release Kinetics of Gefitinib-Loaded PLGA Microspheres. Mol. Pharmaceutics 2017, 14, 459–467. 10.1021/acs.molpharmaceut.6b00896. 27973854

[ref34] Xu Q.; Hashimoto M.; Dang T. T.; Hoare T.; Kohane D. S.; Whitesides G. M.; Langer R.; Anderson D. G. Preparation of Monodisperse Biodegradable Polymer Microparticles Using a Microfluidic Flow-Focusing Device for Controlled Drug Delivery. Small 2009, 5, 1575–1581. 10.1002/smll.200801855. 19296563 PMC2789598

[ref35] Albertini B.; Bertoni S.; Perissutti B.; Passerini N. An Investigation into the Release Behavior of Solid Lipid Microparticles in Different Simulated Gastrointestinal Fluids. Colloids Surf., B 2019, 173, 276–285. 10.1016/j.colsurfb.2018.09.056. 30300834

[ref36] Samouillan V.; Delaunay F.; Dandurand J.; Merbahi N.; Gardou J.-P.; Yousfi M.; Gandaglia A.; Spina M.; Lacabanne C. The Use of Thermal Techniques for the Characterization and Selection of Natural Biomaterials. J. Funct. Biomater. 2011, 2, 230–248. 10.3390/jfb2030230. 24956305 PMC4030942

[ref37] Mohammad M. A.; Grimsey I. M.; Forbes R. T. Mapping the Solid-State Properties of Crystalline Lysozyme during Pharmaceutical Unit-Operations. J. Pharm. Biomed. Anal. 2015, 114, 176–183. 10.1016/j.jpba.2015.05.011. 26068908

[ref38] Elkordy A. A.; Forbes R. T.; Barry B. W. Study of Protein Conformational Stability and Integrity Using Calorimetry and FT-Raman Spectroscopy Correlated with Enzymatic Activity. Eur. J. Pharm. Sci. 2008, 33, 177–190. 10.1016/j.ejps.2007.11.002. 18207710

[ref39] Bresson S.; El Marssi M.; Khelifa B. Raman Spectroscopy Investigation of Various Saturated Monoacid Triglycerides. Chem. Phys. Lipids 2005, 134, 119–129. 10.1016/j.chemphyslip.2004.12.009. 15784230

[ref40] Kagan M. R.; McCreery R. L. Reduction of Fluorescence Interference in Raman Spectroscopy via Analyte Adsorption on Graphitic Carbon. Anal. Chem. 1994, 66, 4159–4165. 10.1021/ac00095a008.

[ref41] Barth A. Infrared Spectroscopy of Proteins. Biochim. Biophys. Acta, Bioenerg. 2007, 1767, 1073–1101. 10.1016/j.bbabio.2007.06.004. 17692815

[ref42] Greenfield N. J. Using Circular Dichroism Spectra to Estimate Protein Secondary Structure. Nat. Protoc. 2006, 1, 2876–2890. 10.1038/nprot.2006.202. 17406547 PMC2728378

[ref43] Kochhar J. S.; Zou S.; Chan S. Y.; Kang L. Protein Encapsulation in Polymeric Microneedles by Photolithography. Int. J. Nanomed. 2012, 7, 3143–3154. 10.2147/IJN.S32000. PMC3392142 22787403

[ref44] Ghisaidoobe A. B. T.; Chung S. J. Intrinsic Tryptophan Fluorescence in the Detection and Analysis of Proteins: A Focus on Förster Resonance Energy Transfer Techniques. Int. J. Mol. Sci. 2014, 15, 22518–22538. 10.3390/ijms151222518. 25490136 PMC4284722

[ref45] Yekta R.; Dehghan G.; Rashtbari S.; Sheibani N.; Moosavi-Movahedi A. A. Activation of Catalase by Pioglitazone: Multiple Spectroscopic Methods Combined with Molecular Docking Studies. J. Mol. Recognit. 2017, 30, e2648 10.1002/jmr.2648. 28626866

[ref46] Pal S.; Dey S. K.; Saha C. Inhibition of Catalase by Tea Catechins in Free and Cellular State: A Biophysical Approach. PLoS One 2014, 9, e102460 10.1371/journal.pone.0102460. 25025898 PMC4099323

[ref47] Rawat S.; Kohli N.; Suri C. R.; Sahoo D. K. Molecular Mechanism of Improved Structural Integrity of Protein in Polymer Based Microsphere Delivery System. Mol. Pharmaceutics 2012, 9, 2403–2414. 10.1021/mp2004065. 22724678

[ref48] Samejima T.; Kamata M.; Shibata K. Dissociation of Bovine Liver Catalase at Low PH. J. Biochem. 1962, 51, 181–187. 10.1093/oxfordjournals.jbchem.a127518. 14496577

[ref49] Prajapati S.; Bhakuni V.; Babu K. R.; Jain S. K. Alkaline Unfolding and Salt-Induced Folding of Bovine Liver Catalase at High PH. Eur. J. Biochem. 1998, 255, 178–184. 10.1046/j.1432-1327.1998.2550178.x. 9692917

[ref50] Jones M. N.; Manley P.; Midgley P. J. W.; Wilkinson A. E. Dissociation of Bovine and Bacterial Catalases by Sodium N-Dodecyl Sulfate. Biopolymers 1982, 21, 1435–1450. 10.1002/bip.360210712. 7115898

[ref51] Deisseroth A.; Dounce A. L. Nature of the Change Produced in Catalase by Lyophilization. Arch. Biochem. Biophys. 1967, 120, 671–692. 10.1016/0003-9861(67)90533-4.

[ref52] Prakash K.; Prajapati S.; Ahmad A.; Jain S. K.; Bhakuni V. Unique Oligomeric Intermediates of Bovine Liver Catalase. Protein Sci. 2002, 11, 46–57. 10.1110/ps.20102. 11742121 PMC2368769

[ref53] Chang L.; Pikal M. J. Mechanisms of Protein Stabilization in the Solid State. J. Pharm. Sci. 2009, 98, 2886–2908. 10.1002/jps.21825. 19569054

[ref54] Moorthy B. S.; Iyer L. K.; Topp E. M. Characterizing Protein Structure, Dynamics and Conformation in Lyophilized Solids. Curr. Pharm. Des. 2015, 21, 5845–5853. 10.2174/1381612821666151008150735. 26446463 PMC4671836

[ref55] Lee S. L.; Hafeman A. E.; Debenedetti P. G.; Pethica B. A.; Moore D. J. Solid-State Stabilization of α-Chymotrypsin and Catalase with Carbohydrates. Ind. Eng. Chem. Res. 2006, 45, 5134–5147. 10.1021/ie0513503.

[ref56] Raufman J. Pepsin. In Encyclopedia of Gastroenterology; Johnson L. R., Ed.; Academic Press: Amsterdam, 2004; Vol. 3, pp 147–148.

[ref57] Koennings S.; Berié A.; Tessmar J.; Blunk T.; Goepferich A. Influence of wettability and surface activity on release behavior of hydrophilic substances from lipid matrices. J. Controlled Release 2007, 119, 173–181. 10.1016/j.jconrel.2007.02.008. 17412444

[ref58] Prajapati H. N.; Dalrymple D. M.; Serajuddin A. T. A comparative evaluation of mono-, di- and triglyceride of medium chain fatty acids by lipid/surfactant/water phase diagram, solubility determination and dispersion testing for application in pharmaceutical dosage form development. Pharm. Res. 2012, 29, 285–305. 10.1007/s11095-011-0541-3. 21861203 PMC3246583

[ref59] Jenning V.; Gohla S. Comparison of wax and glyceride solid lipid nanoparticles (SLN). Int. J. Pharm. 2000, 196, 219–222. 10.1016/S0378-5173(99)00426-3. 10699722

