# Supplementary material for: Solid Lipid Microparticles for Oral Delivery of Catalase: Focus on the Protein Structural Integrity and Gastric Protection
Source: Mol Pharm. 2020 Jul 30;17(9):3609–21. doi: 10.1021/acs.molpharmaceut.0c00666 (PMC8009523; doi:10.1021/acs.molpharmaceut.0c00666)
Supplement: Supplementary file 1 — mp0c00666_si_001.pdf [file mp0c00666_si_001.pdf]

Solid lipid microparticles for oral delivery of catalase: focus on the protein structural integrity and gastric protection

Serena Bertoni,[†] Daniele Tedesco,[‡] Manuela Bartolini,[‡] Cecilia Prata,[§] Nadia Passerini,[†] Beatrice Albertini[†]*

[†]PharmTech Lab, Department of Pharmacy and Biotechnology, University of Bologna, Via S. Donato 19/2, 40127 Bologna, Italy.

[‡]Bio-Pharmaceutical Analysis Section (Bio-PhASe), Department of Pharmacy and Biotechnology, University of Bologna, Via Belmeloro 6, 40126 Bologna, Italy.

[§]Department of Pharmacy and Biotechnology, University of Bologna, Via Irnerio 48, 40126 Bologna, Italy.

Contents

Yellow complex between H_2O_2 and ammonium molybdate……..…………………………..…..……2

Free CAT characterization……………...……………………………..……...……………..……..…3

DSC analysis of free CAT……………………………..…………………………..………..……..…4

Additional DSC analysis of F2, F3 and F4 formulations……………………………………..……5

Raman analysis of SLMs ……………………………………………………………………....……6

FT-IR analysis of SLMs…………………………………………………………………...……6

References…………………………………………………………………..……………….……..…7

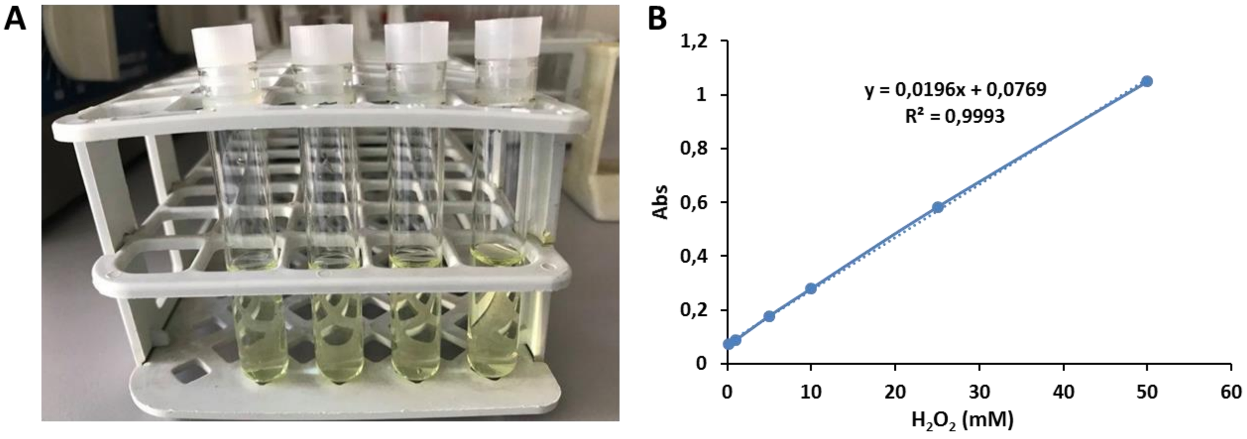

Figure 1 SI. Appearance of the yellow complex formed upon activity assay (A) and calibration curve of the complex at 410 nm (B).

Free CAT characterization.

Kinetic analysis. The kinetic analysis was performed using a direct kinetic spectrophotometric assay based on the decrease in absorption of H_2O_2. 0.3 mL of enzyme solution were added to 1.2 mL of H_2O_2 solution and the initial reaction rates were determined spectrophotometrically by measuring the decrease of absorbance at 240 nm.[1] Different substrate concentrations (H_2O_2 solutions ranging from 5 to 100 mM) were used. Michaelis constants (K_M) and the maximum reaction rate (V_{max}) were calculated from the experimental data using Lineweaver-Burk plots.

Effect of pH and temperature on CAT activity. The effect of pH on enzyme activity was studied performing the activity assay in different buffer solutions. Aliquots of enzyme solution (0.1 mL) were added to 0.4 mL of buffer at different pH values (1, 2, 4, 6, 7 and 9). The effect of temperature on CAT activity was examined by incubating the enzyme solution for 5 min at different temperatures (25, 40, 50, 60, and 80 °C) before performing the activity assay. The results of pH, temperature of the medium are presented in a normalized form, with the highest value of each set being assigned the value of 100% activity.

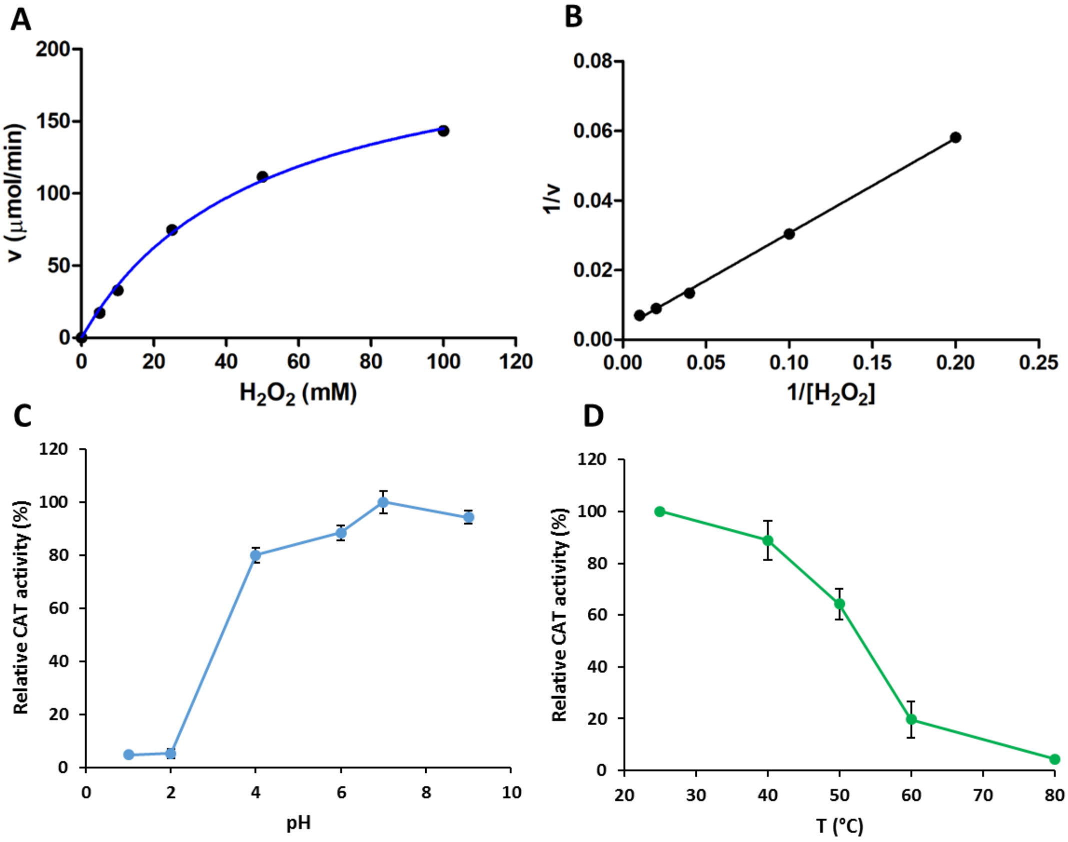

Figure 2 SI. Kinetic analysis of CAT: (A) Michaelis-Menten and (B) Lineweaver-Burk plots. Effect of pH (C) and temperature (D) on CAT activity. Values are expressed as mean (n = 3) ± SD

Results from the kinetic analysis of CAT are reported in Figure 2 SI. The velocity of the reaction increased by increasing the concentration of the substrate, namely H_2O_2, in agreement with Michaelis-Menten kinetics. Data were fitted in a Lineweaver-Burk plot (R_2 = 0.9991) and the estimated kinetic parameters K_M and V_{max} were 49.5 mM and 216.8 µmol min^{-1}, respectively. The high value of K_M, in good agreement with the values reported in the literature,[2,3,4] indicates that the affinity for the substrate is low, and thus a high amount of substrate is needed to saturate the enzyme. CAT has been recognized as an enzyme with a rapid turnover rate.[5] The observed maximum velocity per mg protein corresponded to 4.3 x 10^4 U mg protein-1, in accordance with values already reported[6,7].

The influence of pH and temperature on CAT activity was then investigated. CAT was active in the range of pH between 4 and 9 with an optimum activity at pH 7 (Figure 2 SI, c), in agreement with previous reports.[8, 9] At pH values equal or lower than 2 the enzyme was completely inactive. Denaturation of CAT at acidic pH is the result of the dissociation of the native tetrameric enzyme into dimers, which are enzymatically inactive.[10] The investigation of the thermal stability of CAT (Figure 2 SI, d) showed that the enzyme activity decreased at increasing temperature and dropped dramatically above 40 °C.

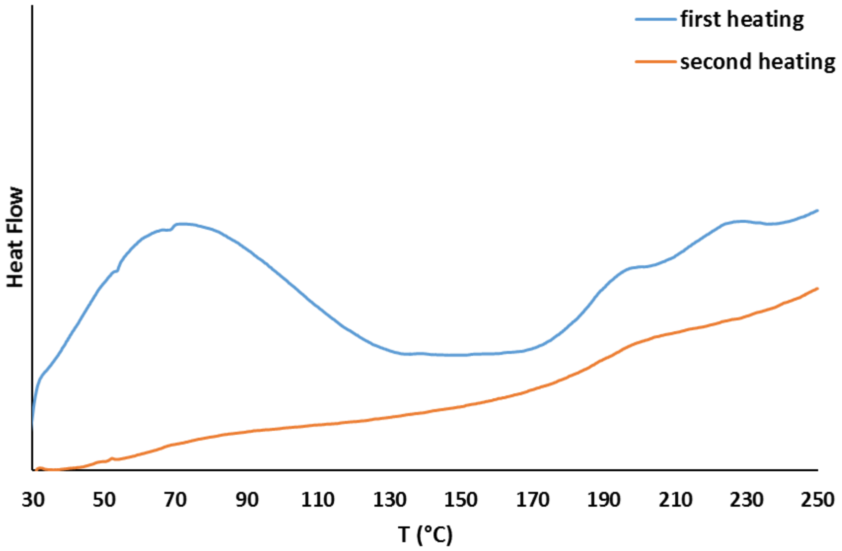

Figure 3 SI. DSC analysis of free CAT performed between 30 and 250 °C in two following heating scans.

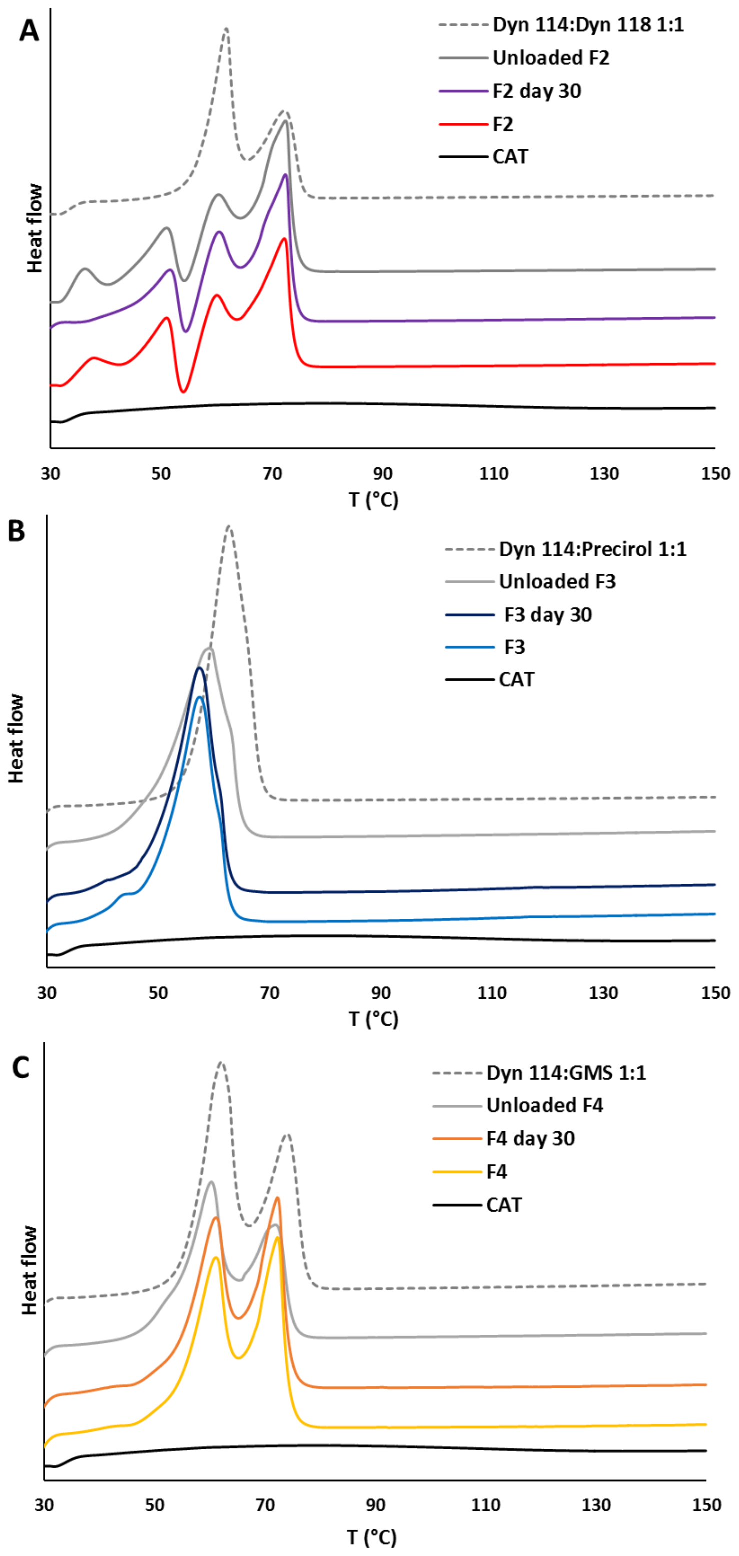

Figure 4 SI. DSC analysis of F2 (A), F3 (B) and F4 (C) formulations. DSC were performed on unloaded SLMs, CAT-loaded F1 immediately after preparation and after 1 month of storage, and compared with free CAT and with the simple carrier powder corresponding to the carrier composition of the SLMs (physical mixture).

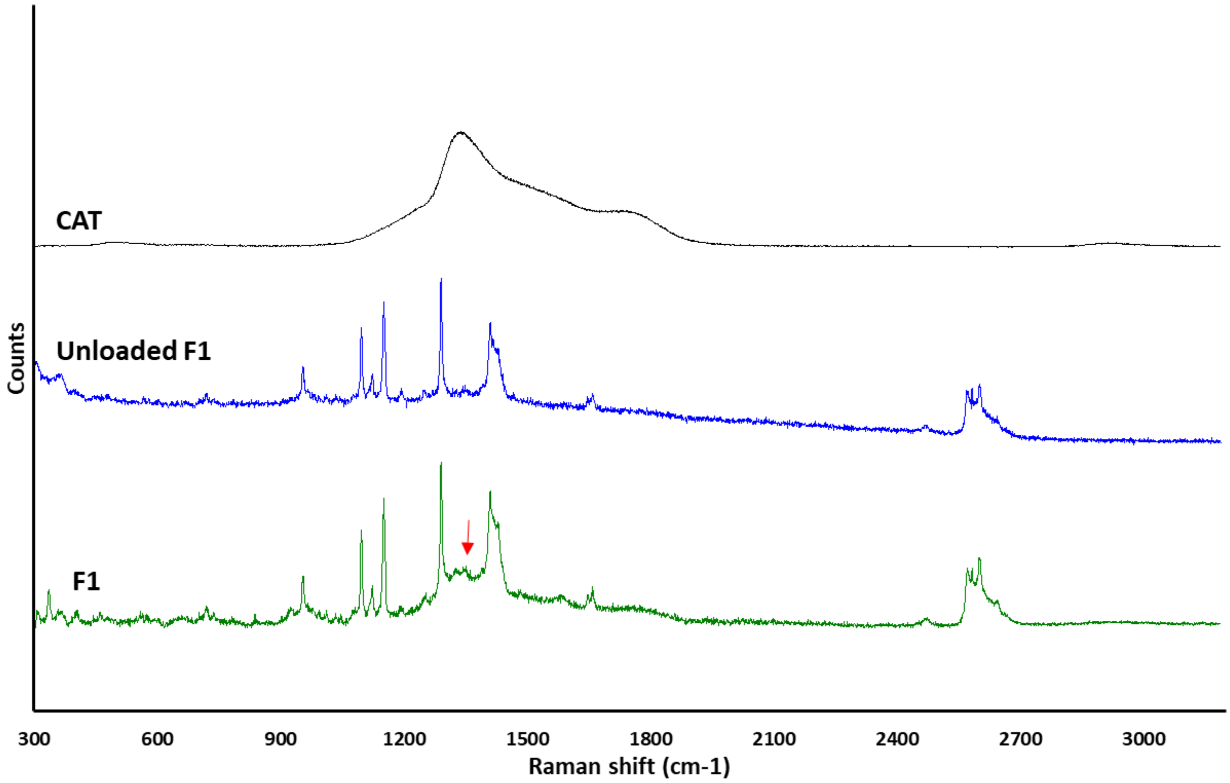

Figure 5 SI. Raman spectra of CAT, unloaded F1 SLMs and CAT-loaded F1 SLMs.

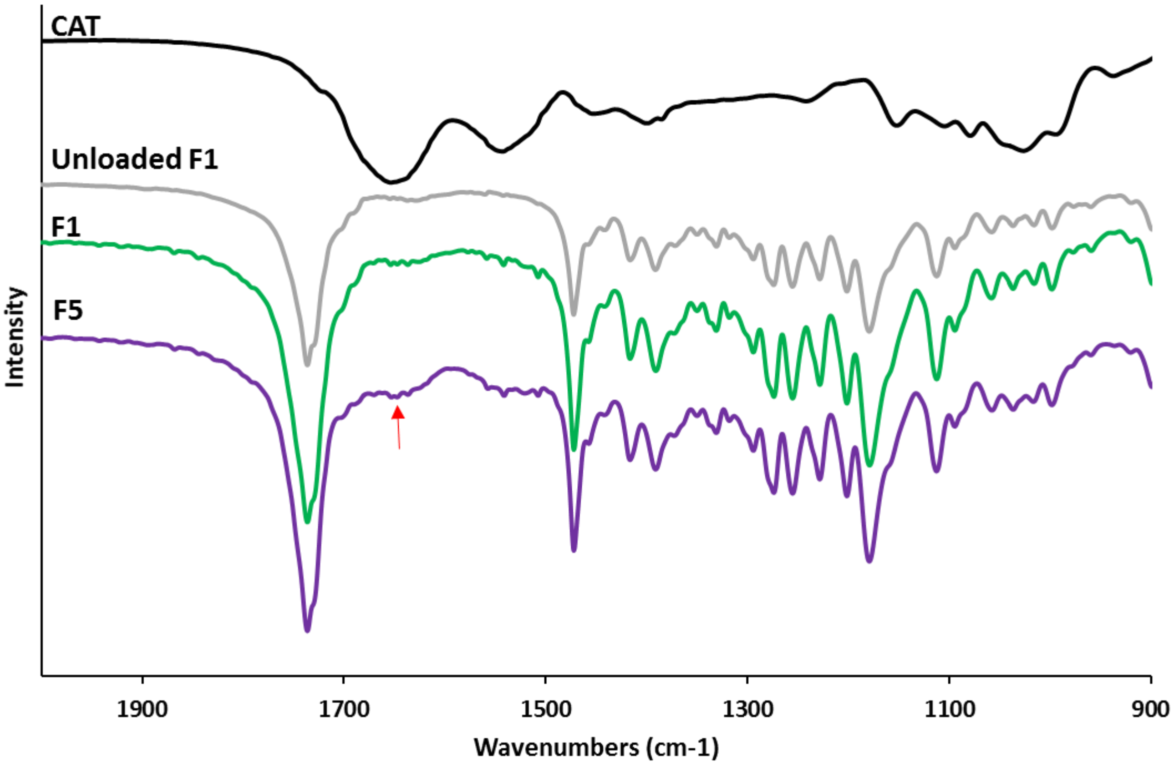

Figure 6 SI. FT-IR spectra of CAT, unloaded F1 SLMs and F1 SLMs loaded with CAT at 5% (F1) and 20% (F5).

References

(1) Beers, R. F.; Sizer, I. W. A Spectrophotometric Method for Measuring the Breakdown of Hydrogen Peroxide by Catalase. *J. Biol. Chem*. **1952**, *195* (1), 133–140.

(2) Darr, D.; Fridovich, I. Inhibition of catalase by 3,3'-diaminobenzidine. *Biochem J.* **1985,** *226* (3), 781–787.

(3) Özlem, A.; Seyhan, T. S.; Deniz, Y. Immobilization and characterization of bovine liver catalase on eggshell. *J. Serb. Chem. Soc*. **2008**, *73* (6), 609-618.

(4) Rashtbari, S.; Dehghan, G.; Yekta R.; Jouyban, A. Investigation of the Binding Mechanism and Inhibition of Bovine Liver Catalase by Quercetin: Multi-spectroscopic and Computational Study. *Bioimpacts* **2017**, *7* (3), 147-153.

(5) Switala, J.; Loewen, P. C. Diversity of properties among catalases. *Arch. Biochem. Biophys*. 2002, 401 (2), 145-154.

(6) J Kaushal, J.; Seema; Singh, G.; Arya, S. K. Immobilization of Catalase onto Chitosan and Chitosan–bentonite Complex: A Comparative Study. *Biotechnol. Reports* **2018**, *18,* e00258.

(7) Çetinus, S. A.; Öztop, H. N. Immobilization of catalase into chemically crosslinked chitosan beads. *Enzyme Microb. Tech*. **2003**, *32* (7), 889-894.

(8) Abdel-mageed, H. M.; Fahmy, A. S.; Shaker, D. S.; Saleh, A. Development of Novel Delivery System for Nanoencapsulation of Catalase : Formulation , Characterization , and in

Vivo Evaluation Using Oxidative Skin Injury Model. *Artif. Cells, Nanomedicine, Biotechnol*. **2018**, *46,* 362–371.

(9) Yoshimoto, M.; Sakamoto, H.; Shirakami, H. Covalent Conjugation of Tetrameric Bovine Liver Catalase to Liposome Membranes for Stabilization of the Enzyme Tertiary and Quaternary Structures. *Colloids Surf. B. Biointerfaces* **2009**, *69* (2), 281–287.

(10) Samejima, T.; Yang, J. T. Reconstitution of Acid-Denaurated Catalase. *J. Biol. Chem*. **1963**, *238*, 3256–3261.
